# Visceral Leishmaniasis on the Indian Subcontinent: Modelling the Dynamic Relationship between Vector Control Schemes and Vector Life Cycles

**DOI:** 10.1371/journal.pntd.0004868

**Published:** 2016-08-18

**Authors:** David M. Poché, William E. Grant, Hsiao-Hsuan Wang

**Affiliations:** Department of Wildlife and Fisheries Sciences, Texas A&M University, College Station, Texas, United States of America; Imperial College London, Faculty of Medicine, School of Public Health, UNITED KINGDOM

## Abstract

**Background:**

Visceral leishmaniasis (VL) is a disease caused by two known vector-borne parasite species (*Leishmania donovani*, *L*. *infantum*), transmitted to man by phlebotomine sand flies (species: *Phlebotomus* and *Lutzomyia*), resulting in ≈50,000 human fatalities annually, ≈67% occurring on the Indian subcontinent. Indoor residual spraying is the current method of sand fly control in India, but alternative means of vector control, such as the treatment of livestock with systemic insecticide-based drugs, are being evaluated. We describe an individual-based, stochastic, life-stage-structured model that represents a sand fly vector population within a village in India and simulates the effects of vector control via fipronil-based drugs orally administered to cattle, which target both blood-feeding adults and larvae that feed on host feces.

**Principle findings:**

Simulation results indicated efficacy of fipronil-based control schemes in reducing sand fly abundance depended on timing of drug applications relative to seasonality of the sand fly life cycle. Taking into account cost-effectiveness and logistical feasibility, two of the most efficacious treatment schemes reduced population peaks occurring from April through August by ≈90% (applications 3 times per year at 2-month intervals initiated in March) and >95% (applications 6 times per year at 2-month intervals initiated in January) relative to no control, with the cumulative number of sand fly days occurring April-August reduced by ≈83% and ≈97%, respectively, and more specifically during the summer months of peak human exposure (June-August) by ≈85% and ≈97%, respectively.

**Conclusions:**

Our model should prove useful in *a priori* evaluation of the efficacy of fipronil-based drugs in controlling leishmaniasis on the Indian subcontinent and beyond.

## Author Summary

Visceral leishmaniasis is a disease caused by a virulent vector-borne parasite transmitted to man by phlebotomine sand flies. Fipronil-based drugs, administered to cattle orally, provide a potential means of sand fly control by permeating in cattle blood and being excreted in cattle feces, targeting adult females feeding on cattle blood and larvae feeding on cattle feces, respectively. An agent-based, stochastic simulation model was developed to represent sand fly population dynamics in a village in Bihar, India, at all developmental stages, with the goal of predicting the impact of various vector control strategies, utilizing drug treated cattle, on vector population numbers. Results indicate that success of treatment is dependent on the number of treatments applied annually and the seasonality of the sand fly lifecycle. Results further suggest that treatment schemes are most effective in reducing vector populations when high drug efficacy is maintained in cattle feces during periods of high larval density. Our approach incorporates detailed representation of the vector population and provides an explicit representation of the effects of insecticide application on adult and larval sand flies. Hence, this model predicts treatment schemes that may have the greatest potential to reduce sand fly numbers.

## Introduction

The deadliest form of leishmaniasis, visceral leishmaniasis (VL), is vector-transmitted through the bite of phlebotomine sand flies in the *Phlebotomus* and *Lutzomyia* genera. This protozoan parasite results in an estimated 500,000 human infections and 50,000 human fatalities annually, making it the second most prevalent parasitic killer on Earth, behind only malaria [1,2]. The highest global rate of occurrence is on the Indian subcontinent with approximately 67% of all human instances occurring in India, Bangladesh and Nepal in areas of extreme poverty and high population density [3]. Bihar is the most impoverished, most densely populated, and most VL-endemic state in India, with 90% of the Indian VL cases reported there [4]. The VL pathogen, *Leishmania donovani*, is described as anthroponotic on the Indian subcontinent with humans acting as the only known reservoir for infection [5].

### Vector ecology

The known VL vector on the Indian subcontinent is the sand fly species *Phlebotomus argentipes* [6]. Phlebotomine sand flies are small Diptera, rarely exceeding a length of 3 mm, in the family Psychodidae and subfamily Phlebotominae [7] and are holometabolous consisting of four life stages: eggs, larvae, pupae, and adults. Sand flies are active primarily at night and are regarded as silent feeders [8]. *P*. *argentipes* females host blood feed primarily on cattle and humans within rural villages [9–12]. The blood meal is required in order to complete the oviposition process. Immature sand flies in Bihar have been found largely in areas within and surrounding cattle sheds [13–15], suggesting cattle feces may serve as a food source for larvae which feed on organic matter. Results of several laboratory experiments have found sand fly processes such as development, mortality and reproduction to be temperature-dependent with many of these processes occurring more rapidly at higher temperatures [16–20]. Nightly air temperatures in Bihar will exceed 20°C between March-October and are highest during the summer (June-August) and the observed sand fly population is small in January and February when minimum temperatures are lowest [21].

### Vector control

It has been suggested that further research regarding alternative or integrated vector control approaches should be examined to supplement the current practice [22]. Vector control in India comes in the form of indoor residual spraying (IRS) performed historically with DDT and more recently with synthetic pyrethroids. IRS controls endophilic sand flies, but blood-fed sand flies have been collected outdoors and indoors [9,21,23]. A survey concluded that roughly 95% of Bihari villager households have family members that sleep outdoors at least part of the year [24]. Logically, these villagers are therefore not protected by IRS and are potentially exposed to exophilic sand flies.

Fipronil-based drugs, orally administered to cattle and rodents, have been successful in killing laboratory-reared sand flies under controlled conditions, targeting blood-feeding adults and larvae that feed on host feces [25–27]. Orally applied fipronil can remain in the system of animals for several weeks to several months, dependent on the concentration administered (mg/kg body weight) and fipronil has a lengthy half-life of approximately 128 days [28], meaning that sand fly control can potentially be maintained for several months following a single treatment. With this form of treatment, the success of vector control could be independent of exophilic or endophilic feeding behavior and be dependent on host and oviposition site preferences. Hence, this form of treatment could potentially supplement the current practice of IRS by targeting exophilic, cattle-feeding adult sand flies and larval sand flies feeding on organic matter in the form of cattle feces.

### Evaluating control success

A reduction in vector density should lead to a reduction in the transmission rate of VL as suggested by a recent VL model which predicted that either reducing vector density >67% through application of adulticides or >79% through breeding site destruction could eliminate the ability of the VL pathogen to persist [29]. Vector and pathogen seasonality in addition to social practice should be taken into consideration when developing a control plan. Not only should overall vector density be considered, but one also should consider vector density during spring/summer months (April-August) when villagers could potentially be at greatest risk of exposure to infected sand flies. Clinical VL in Bihar is commonly reported between the months of April and August [30]. Proper bed net usage during the warmer months of the year has been found to be strongly protective against VL [31]. However, several publications suggest that bed net usage in Asia and Africa declines in response to increased temperature [32–37].

### Mathematical modelling

Susceptible-Infected-Recovered (SIR) compartment models, and variants of this, have been developed in the past to represent VL epidemiology within human populations on the Indian subcontinent. The first such model examined three historical VL epidemic peaks in Assam, India which occurred between 1875 and 1950 and concluded that intrinsic processes related to host and vector dynamics, rather than extrinsic factors such as earthquakes or influenza outbreaks, provided the simplest explanation of the timing of the peaks [38]. More recent models representing VL epidemiology within human populations in Bihar have examined VL underreporting [39], antimony resistant VL [40], VL treatment, prevention, and control [41], and more specific vector control strategies, namely the application of adulticides and destruction of sand fly breeding sites [29]. The latter model represented the application of adulticides and the destruction of sand fly breeding sites via variables that reduced sand fly life expectancy and breeding site capacity, respectively, and predicted the impact of reducing vector density on the ability of the pathogen to persist (as indicated by the basic reproduction number R_o_ [42]).

The model originally published by [40] and subsequently used in [39] and [29] is a deterministic SIR-type model that focuses heavily on the natural history of VL infection within human populations, represented by 11 distinct stages. However, the vector (sand fly) population is represented by only three stages: the *susceptible*, *latent*, and *infectious*, with abundance of the latter used to calculate the VL transmission rate to humans. Emergence rate of susceptibles and mortality rates of each stage are held constant. The egg, larval, and pupal stages of the sand fly life cycle are not represented in this model, or in any other SIR-type VL model to the best of our knowledge. These limitations to current SIR-type models have been recognized and the exploration of individual-based, stage-structured, stochastic modelling approaches has been recommended [29], which could allow explicit evaluation of stage-specific impacts of vector control strategies on sand fly populations in Bihar.

### Objectives

In this paper we describe an individual-based, stochastic, stage-structured model that represents a temperature-driven sand fly vector population within a village in Bihar, India and simulates the effects of vector control through the use of fipronil-based drugs orally administered to cattle. The model does not include a human population or VL pathogen, but rather focuses on the effects of fipronil-induced mortality of larval and adult life stages on sand fly population dynamics. We first describe the model and evaluate its performance. We then use the model to simulate several fipronil-based control schemes in which we vary treatment frequency and timing of treatment application, focusing on resulting reductions in sand fly populations during spring/summer and especially during the period of peak human exposure (June-August). We also examine sensitivity of model predictions of treatment efficacy to parametric uncertainty.

## Materials and Methods

### Model overview

**Fig 1:**
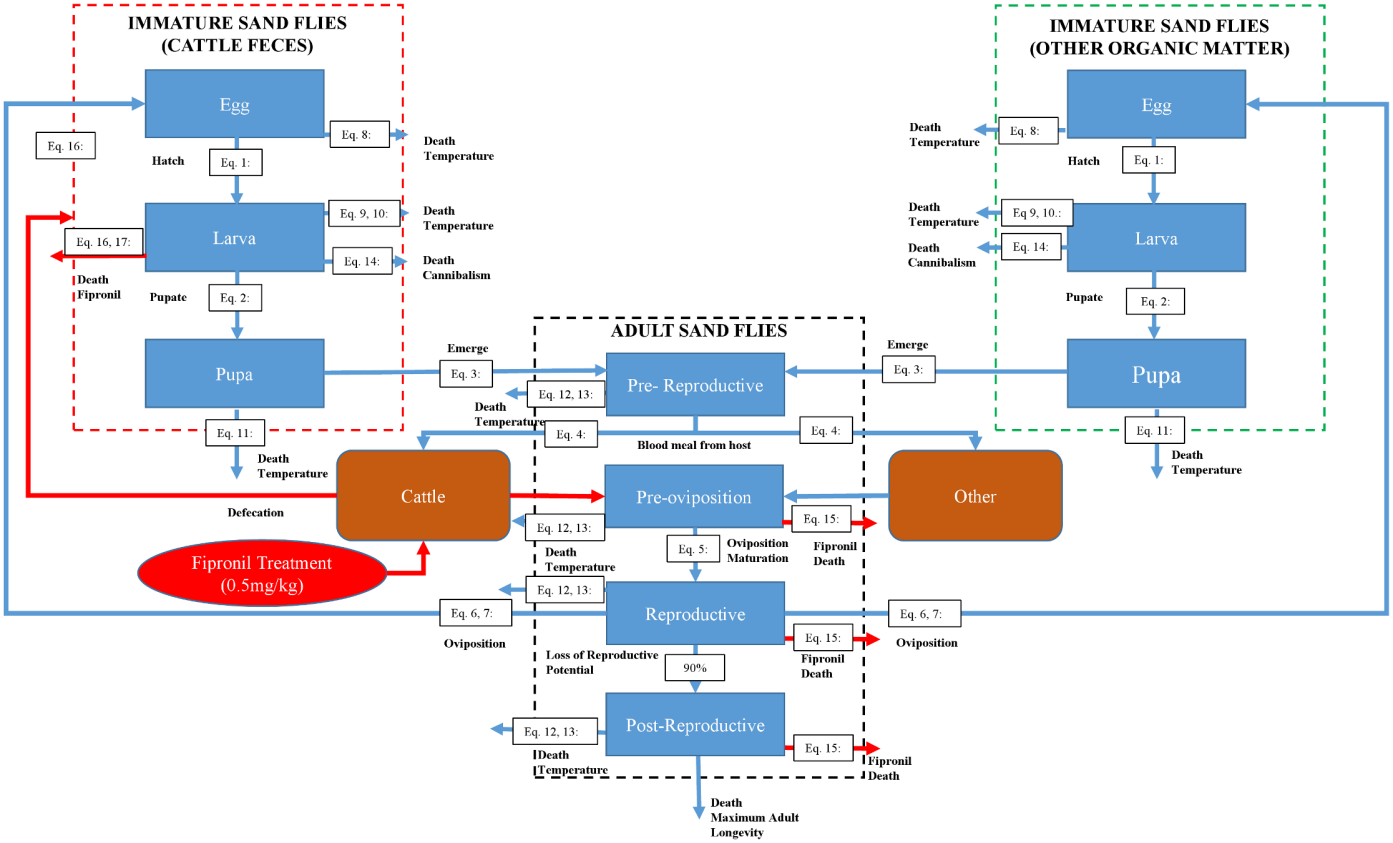
Conceptual model representing the impact of fipronil-induced adult and larval mortality on population dynamics of sand flies. Sand flies are represented as eggs, larvae, pupae, pre-reproductive adults, pre-oviposition adults, reproductive adults, and post reproductive adults. Pre-reproductive adults require a blood meal to proceed with oviposition. Fipronil increases the mortality rate of adults feeding on treated cattle and larvae feeding on feces excreted by treated cattle. All processes represented by eqs 1–3 and 5–13 are temperature dependent.

The model represents the lifecycle of sand flies as they develop from eggs to larvae to pupae to pre-reproductive adults to pre-oviposition adults to reproductive adults to post-reproductive adults, as well as fipronil-induced larval and adult mortality (Fig 1). Rates of development, natural mortality, and reproduction depend on the environmental temperatures to which the sand flies are exposed. Eggs, larvae, and pupae are exposed to temperatures of the organic matter in which they develop, whereas adults are exposed to ambient temperatures. Natural mortality of larvae also depends on the density of larvae in the organic matter in which they are feeding. Fipronil-induced mortality occurs in adult flies that obtain a blood meal from fipronil-treated cattle, and in larvae that feed on feces from fipronil-treated cattle. Simulations are run on a daily time step, thus all rates and probabilities described below are calculated on a daily basis. Eggs, larvae, and pupae are represented as daily cohorts whereas adults are represented as individuals. That is, the size of each daily cohort of eggs that enter the system is monitored as these eggs develop into larvae and then into pupae. When a cohort of pupae develops to the adult stage, the resulting adults are represented as individual organisms and are followed through pre-reproductive, pre-oviposition, reproductive, and post-reproductive stages (only adult females are represented in the model).

Below we present the equations used in the model to represent the development, reproduction, natural mortality, and fipronil-induced mortality of sandflies.

### Development

**Fig 2:**
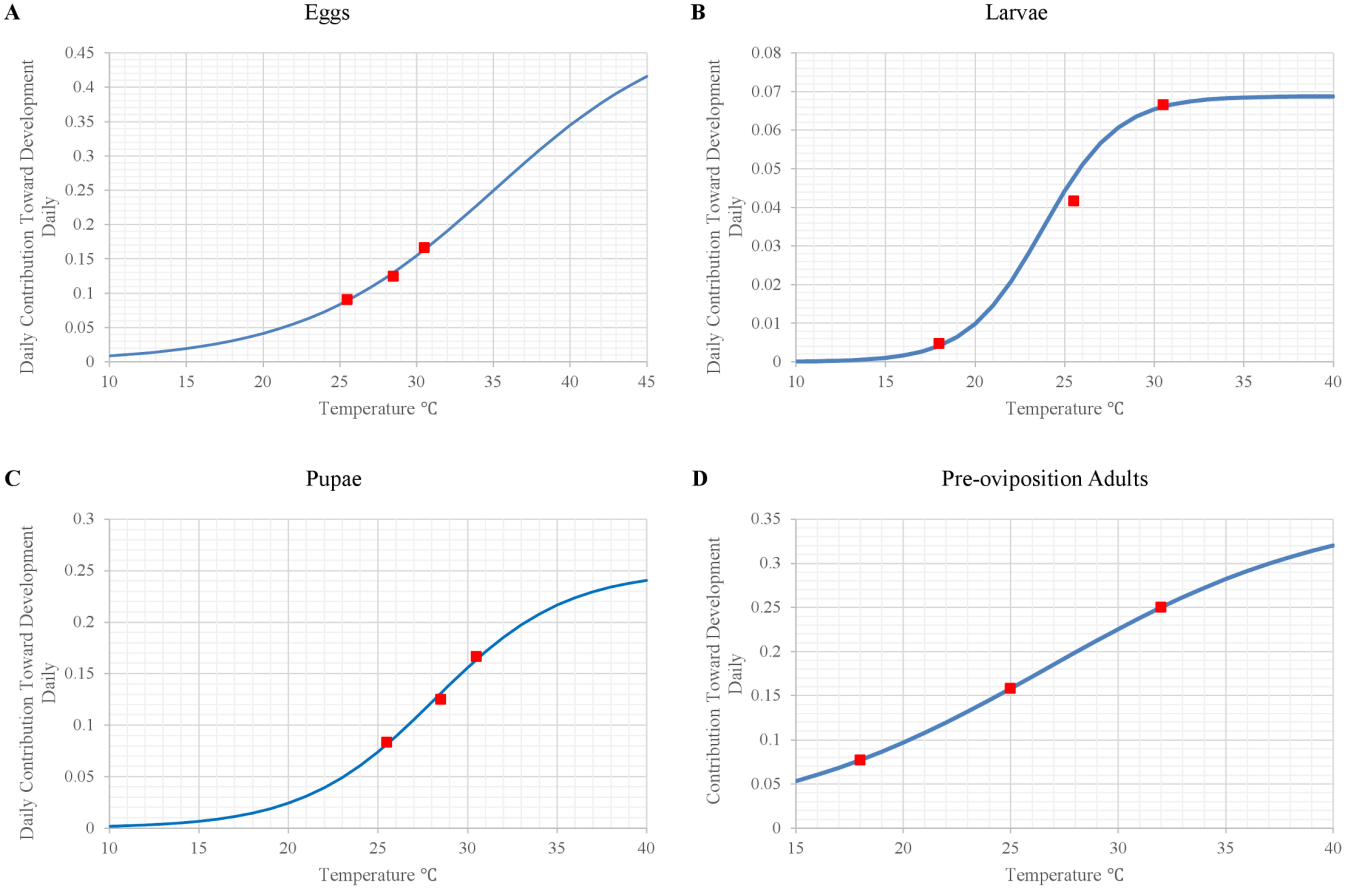
Curves representing of the daily contribution of temperature toward development of sand fly stages. (A) eggs (Eq 1), (B) larvae (Eq 2), (C) pupae (Eq 3), and (D) pre-oviposition adults (Eq 5). Red squares represent the data points used to generate the curves.

To calculate rates of development of immature stages (eggs, larvae, pupae), we drew upon results of laboratory experiments conducted under constant temperatures [16,18,19] and then estimated temperature-dependent development under variable temperature regimes using the general equation described by [43]: 100/*n*_*l*_ = *K*/[1+ *exp*(*a* − *bx*)]. This is a bisymmetrical, sigmoid curve with the distance between the lower and upper developmental temperature thresholds (*K*) estimated as $K=\left[2C_{1}C_{2}C_{3}-C_{2}^{2}\left(C_{1}+C_{3}\right)\right]/\left(C_{1}C_{3}-C_{2}^{2}\right)$, where *C*_*1*_, *C*_*2*_, and *C*_*3*_ are values for 100/*n*_*l*_ on the curve at three temperatures on the abscissa. We represented the temperature-dependent development of eggs, larvae, and pupae as:

$$C_{i,Eggs}=0.5/\left[1+exp\left(-0.1601\cdot T_{i,O}+5.6067\right)\right]$$ (1)

$$C_{i,Larvae}=0.0688052/\left[1+exp\left(-0.4754\cdot T_{i,O}+11.298\right)\right]$$ (2)

$$C_{i,Pupae}=0.25/\left[1+exp\left(-0.2736\cdot T_{i,O}+7.7067\right)\right]$$ (3)

where *C*_*i*,*Eggs*_, *C*_*i*,*Larvae*_, and *C*_*i*,*Pupae*_ represent the contributions of the current daily temperature on day *i* toward the development of eggs, larvae, and pupae, respectively, and *T*_*i*,*O*_ represents current temperature (°C) within the organic matter on day *i* (Fig 2a–2c). The model accumulates *C*_*i*_ over time separately for each cohort, and when Σ_*i*_*C*_*i*_ = 1.0 for a given cohort, the organisms in that cohort advance to the next developmental stage (Fig 1).

**Fig 3:**
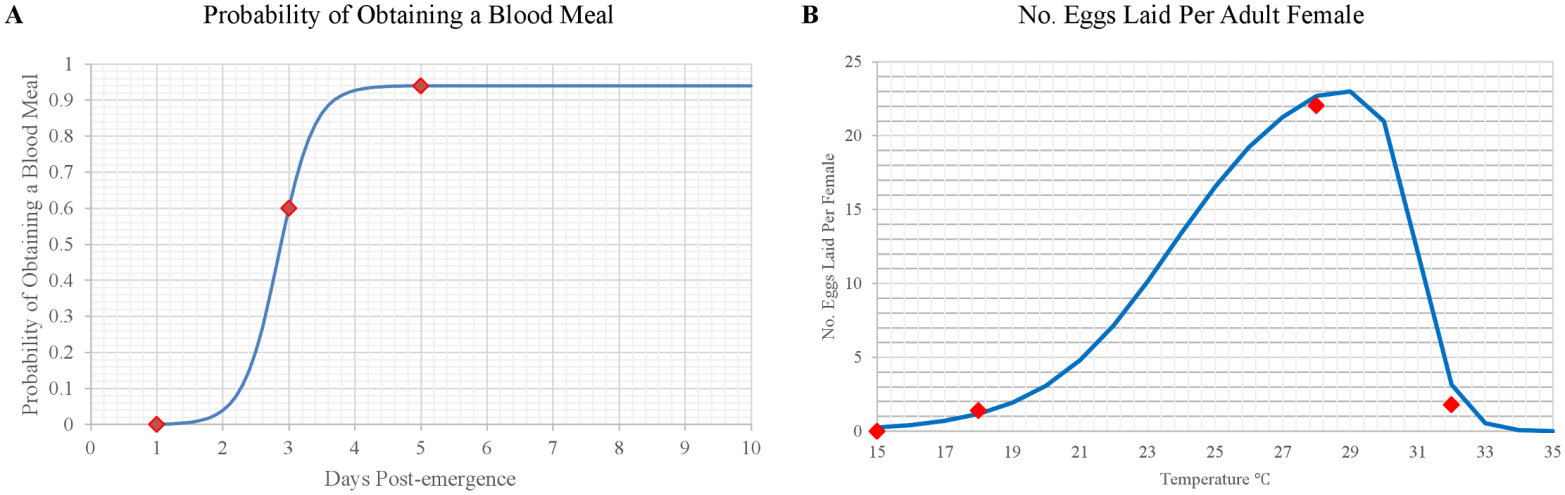
Curves representing (A) the daily probability of obtaining a blood meal as a function of days-post-emergence (Eq 4) and (B) the number of eggs laid per reproductive female as a function of current air temperature (Eqs 6 and 7). Red squares represent the data points used to generate the curves.

After pupation, pre-reproductive adults must obtain a blood meal to advance to the pre-oviposition stage (Fig 1). We estimated the daily probability of obtaining a blood meal based on laboratory experiments in which 0, 3, 60, 85, 94, and 96% of flies obtained their first blood meal by the end of their first, second, third, fourth, fifth, and sixth day, respectively, as an adult [44], and used these results to develop the following curve:

$$P_{i,Blood\;Meal}=0.940321952\;/\left[1+exp\left(-3.7061\cdot D_{i,PE}+10.551\right)\right]$$ (4)

where *P*_*i*,*Blood Meal*_ is the probability of a pre-reproductive adult obtaining a blood meal on day *i* and *D*_*i*,*PE*_ is the number of days-post-emergence from pupation (Fig 3a).

We estimated the temperature-dependent development of adults from the pre-oviposition stage to the reproductive stage based on laboratory data collected by [19] in the same manner as described above for eggs, larvae, and pupae:

$$C_{i,POAdults}=0.363755/\left[1+exp\left(-0.1503\cdot T_{i,A}+4.0206\right)\right]$$ (5)

where *C*_*i*,*POAdults*_ is defined and calculated in the same manner as the analogous terms in Eqs 1 through 3, except that *T*_*i*,*A*_ represents current air temperature (°C) on day *i* rather than temperature within organic matter (Fig 2d). When Σ_*i*_*C*_*i*_ = 1.0, flies advance from the pre-oviposition to the reproductive stage (Fig 1).

### Reproduction

Females lay eggs the day they advance from the pre-oviposition to the reproductive stage. We represented the number of eggs laid per reproductive female (Eqs 6 and 7) as a function of temperature (Fig 3b) based on laboratory observations [19]:

If *T*_*i*,*A*_ ≤ 28.5 then

$$P_{i,OElAdults}=25.1464684/\left[1+exp\left(-0.5238\cdot T_{i,A}+12.441\right)\right]$$ (6)

If *T*_*i*,*A*_ > 28.5 then

$$P_{i,OElAdults}=25.1464684-25.1464684/\left[1+exp\left(-0.5238\cdot T_{i,A}+12.441\right)\right]$$ (7)

where *P*_*i*,*OElAdusts*_ represents the number of eggs laid by a female on day *i* and *T*_*i*,*A*_ represents the current air temperature (°C) on day *i*. However, no eggs are laid if *T*_*i*,*A*_ ≤ 15C [19].

After oviposition, reproductive females have a 90% chance of becoming post-reproductive and a 10% chance of returning to the pre-reproductive stage [17]. If they return to the pre-reproductive stage, the daily probability of obtaining another blood meal is calculated using Eq 4, except *D*_*i*,*PE*_ is redefined as the number of days since returning to the pre-reproductive stage.

### Natural mortality

**Fig 4:**
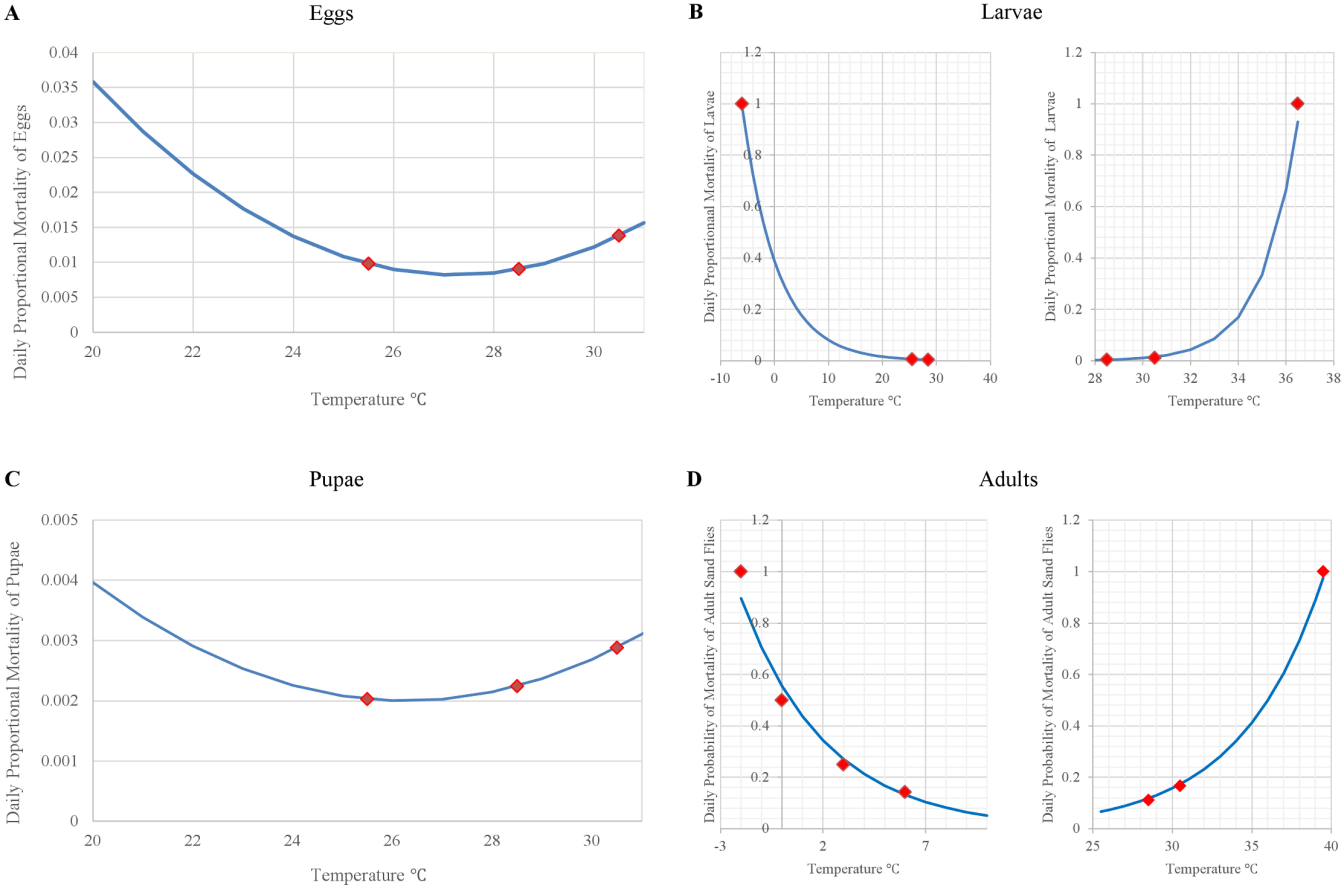
Curves representing the temperature-dependent natural mortality of sand fly stages. (A) eggs (Eq 8), (B) larvae (Eqs 9 and 10), (C) pupae (Eq 11), and (D) adults (Eqs 12 and 13). Egg and pupal mortality are polynomial functional relationships whereas larval and adult mortality increases exponentially from the optimum temperatures for survivorship towards the upper and lower thermal limits. Red squares represent the data points used to generate the curves.

Natural mortality of cohorts of eggs, larvae, and pupae depend on the temperature (*T*_*i*,*O*_) of the organic matter (Fig 4a–4c) in which they are located, whereas natural mortality of adults depends on air temperatures (*T*_*iA*_) (Fig 4d). We represented the temperature-dependent natural mortality of eggs, larvae, pupae, and adults based on laboratory experiments conducted by [16,45]:

$$P_{i,MEggs}=0.00052737\cdot T_{i,O}^{2}-0.02872971\cdot T_{i,O}+0.39946900$$ (8)

If *T*_*i*,*A*_ ≤ 28.5 then

$$P_{i,MlLarvae}=0.3898*\text{exp}\left(-0.156*T_{i,O}\right)$$ (9)

If *T*_*i*,*A*_ > 28.5 then

$$P_{i,MuLarvae}=0.0000000000144*exp\;\left(0.68195*T_{i,O)}\right)$$ (10)

$$P_{i,MPupae}=0.00004973\cdot T_{i,O}^{2}-0.00261400\cdot T_{i,O}+0.03635092$$ (11)

If *T*_*i*,*A*_ ≤ 10 then

$$P_{i,Ml\;Adults}=0.5556*\text{exp}\left(-0.239*T_{iA}\right)$$ (12)

If *T*_*i*,*A*_ > 10 then

$$P_{i,Mu\;Adults}=0.0005*\text{exp}\left(0.1918*T_{iA}\right)$$ (13)

where *P*_*i*,*MEggs*_, *P*_*i*,*MlLarvae*_, *P*_*i*,*MuLarvae*_, *P*_*i*,*MPupae*_ represent the proportion of eggs, larvae, and pupae dying on day *i* and *P*_*i*,*Ml Adults*_ and *P*_*i*,*Mu Adults*_ represent the daily probability of dying for adults on day *i*. Independent of temperature, we estimated the maximum longevity of adults in the wild to be 30 days based on findings presented by [46].

We also represented density-dependent natural mortality of larvae based on rates of cannibalism observed in laboratory experiments conducted with different larval densities [47]:

$$P_{i,MLarvae\_C}=(r_{i,Can})*N_{i,Larvae}$$ (14)

where *P*_*i*,*MLarvae_c*_ represents the proportion of larvae dying on day *i* due to cannibalism, *N*_*i*,*Larvae*_ represents the number of larvae in the system on day *i*, and *r*_*i*,*Can*_ represents a proportional increase in cannibalism as the number of larvae increases. The data presented by [47] suggest an approximately linear relationship between larval density and rate of cannibalism, the slope of which (*r*_*i*,*Can*_) we calibrated, as described in the Model evaluation and Model calibration sections below.

### Fipronil-induced mortality

In addition to depending on the frequency of treatment application and the proportion of the cattle treated, which we represented as management variables, rates of fipronil-induced mortality depend on (1) the proportion of adult sand flies that feed on cattle, (2) the proportion of larvae that feed in organic matter containing cattle feces, (3) the efficacy of fipronil contained in the blood of cattle, and (4) the efficacy of fipronil contained within cattle feces. We assumed that 50% of adult flies obtain their blood meal from cattle [9] and that 90% of eggs are laid on, and hence larvae develop in, organic matter containing cattle feces [13].

**Fig 5:**
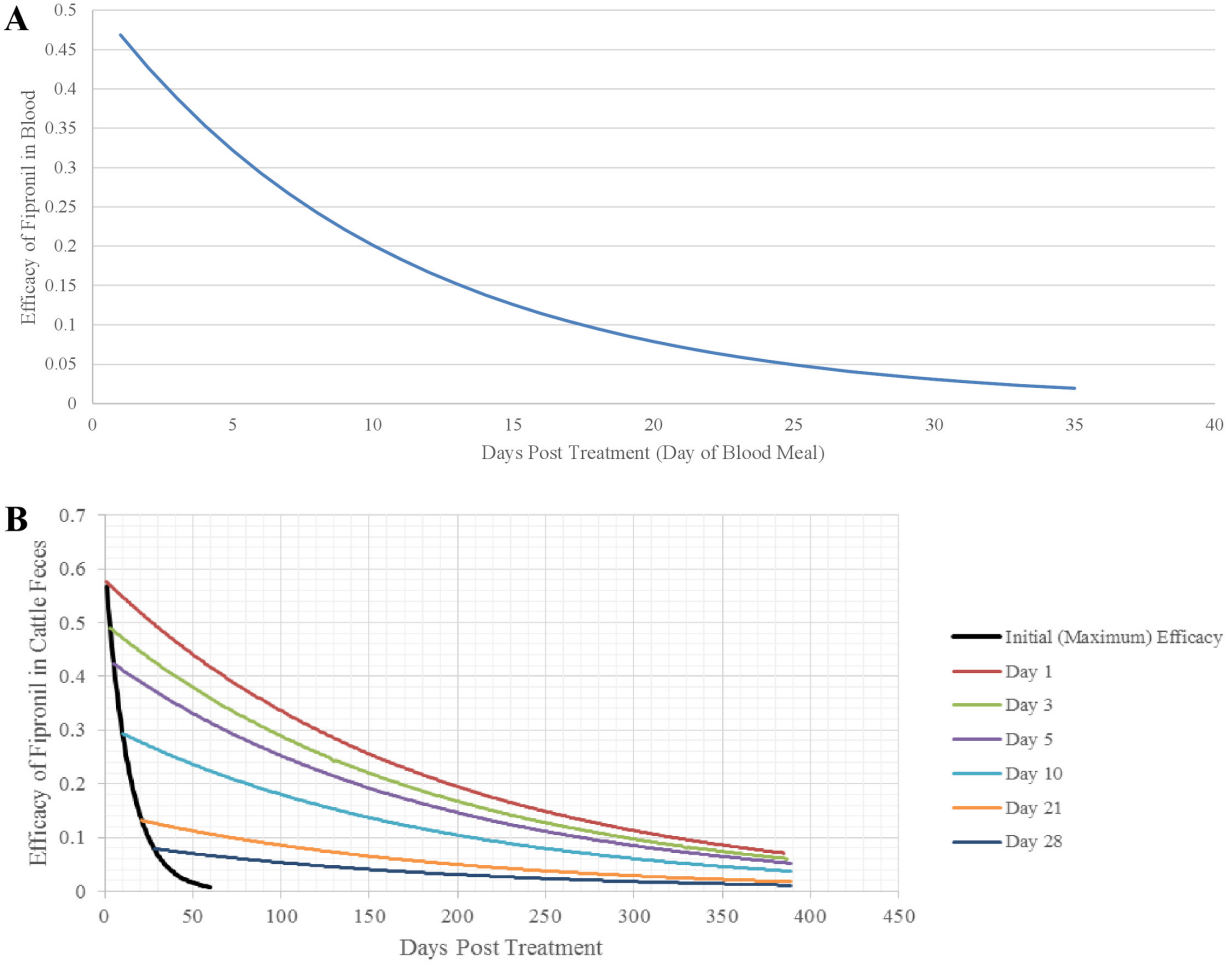
Curve(s) representing fipronil-induced sand fly mortality. (A) the decline in fipronil efficacy (measured as daily probability of mortality of adults) in cattle blood as a function of days post-application (Eq 15) and (B) the decline in fipronil efficacy (measured as daily proportional mortality of larvae) in cattle feces as a function of number of days post-defecation (Eq 16) (colored lines) and the number of days-post-application when defecation occurred (Eq 17) (black line).

We represented the efficacy of fipronil within the blood of cattle as decreasing exponentially as a function of the number of days after fipronil application:

$$P\prime_{i,MAdults}=0.515\;\cdot \text{exp}\left(-0.094\cdot D_{i,PT}\right)$$ (15)

where *P’*_*i*,*MAdults*_ represents the daily probability of dying for an adult fly that obtained a blood meal from treated cattle *D*_*i*,*PT*_ days post-treatment (days after application of fipronil) [25] (Fig 5a). Once an adult obtains a blood meal from a treated cow, we assumed that its daily probability of dying due to fipronil did not change, that is, efficacy of the fipronil within the fly remained constant.

We represented the proportion of larvae dying due to fipronil within cattle feces as decreasing exponentially as a function of the number of days post-defecation [28,48]:

$$P\prime_{i,Larvae}=E_{j}\cdot \text{exp}\left(-0.00545\cdot D_{i,PD}\right)$$ (16)

where *P’*_*i*,*MLarvae*_ represents the proportion of larvae dying on day *i* that are feeding on feces of treated cattle *D*_*i*,*PD*_ days post-defecation (*D*_*i*,*PD*_ days after the feces were deposited), assuming the feces were deposited *j* days after application of fipronil. The initial (maximum) efficacy of fipronil in cattle feces (*E*_*j*_) (Eq 17) itself decreases exponentially over time [25]:

$$E_{j}=0.567\cdot \text{exp}\left[-0.073\left(D_{i,PT}-1\right)\right]$$ (17)

For example, fresh feces deposited 1 day after cattle are treated have a higher efficacy than fresh feces deposited 2 days after cattle are treated (Fig 5b). We assumed that fipronil-induced mortality and natural mortality were completely additive.

### Model evaluation

To evaluate the potential usefulness of the model in simulating the population-level response of sand flies to fipronil-induced mortality, we first verified that the model simulated adequately the rates of development, reproduction, natural mortality, and fipronil-induced mortality observed under laboratory conditions. That is, that the model code produced simulated data that mimicked the laboratory data from which it was parameterized when we simulated the laboratory experiments. We next calibrated the model to represent environmental conditions typical of Bihar, India such that the simulated population established a seasonally-varying, dynamic equilibrium under baseline conditions (without fipronil-induced mortality). We then evaluated performance of the baseline model by (1) assessing the ecological interpretability of seasonal trends in the simulated sand fly life cycle and (2) comparing simulated fluctuations in abundance of adult sand flies to fluctuations observed in each of three villages in Bihar over a 12-month period. We ran 10 replicate stochastic (Monte Carlo) simulations for each portion of the model evaluation procedure, except for the simulations required for verification of the temperature-dependent development and mortality rates of eggs, larvae, and pupae, which were deterministic.

### Simulated responses of sand fly populations to control schemes using fipronil-treated cattle

#### Experimental design

We assessed the potential efficacy of various schemes using fipronil-treated cattle to control sand fly populations by running 20 sets of simulations (10 replicate stochastic (Monte Carlo) simulations per set) in which we varied (1) the frequency of treatment application and (2) the seasonality of treatment application (Table 1). During each simulation, the system was allowed to establish a dynamic equilibrium, without treatment, for 2 years, then treatment was applied annually for 3 consecutive years, during which time the abundance of adult sand flies was monitored. We assessed efficacy of the different schemes based on suppression of adult sand fly population peaks occurring between April-August and June-August during the third year of treatment, as well as reduction in the cumulative number of adult sand fly days occurring during these times. (Ten Monte Carlo simulations of each scheme allowed detection of a difference of 12,875 SFDs among treatments with a Type I error = 0.05 and a Type II error = 0.01, or a difference ≈ 7% of the mean SFDs under the most efficacious treatment.) June-August is the period during which mean minimum (nighttime) air temperatures are highest in Bihar and sand fly density increases dramatically from April-May being maintained at high density [21], suggesting a decline in bed net usage and an increase in the number of individuals sleeping outdoors.

**Table 1:** Experimental design involving 20 treatment schemes reapplied over a three-year period (with 10 replicate stochastic (Monte Carlo) simulations per scheme).

No. treatments	Jan.	Feb.	Mar.	Apr.	May	Jun.	Jul.	Aug.	Sep.	Oct.	Nov.	Dec.
1	X											
1		X										
1			X									
1				X								
1					X							
1						X						
1							X					
1								X				
1									X			
1										X		
1											X	
1												X
3	X		X		X							
3			X		X		X					
3					X		X		X			
3							X		X		X	
3	X								X		X	
6	X		X		X		X		X		X	
6		X		X		X		X		X		X
12	X	X	X	X	X	X	X	X	X	X	X	X

X indicates the month of treatment application. Treatment occurs on the first day of the month.

## Results

### Model verification

#### Development of immatures: Eggs, larvae, pupae

**Fig 6:**
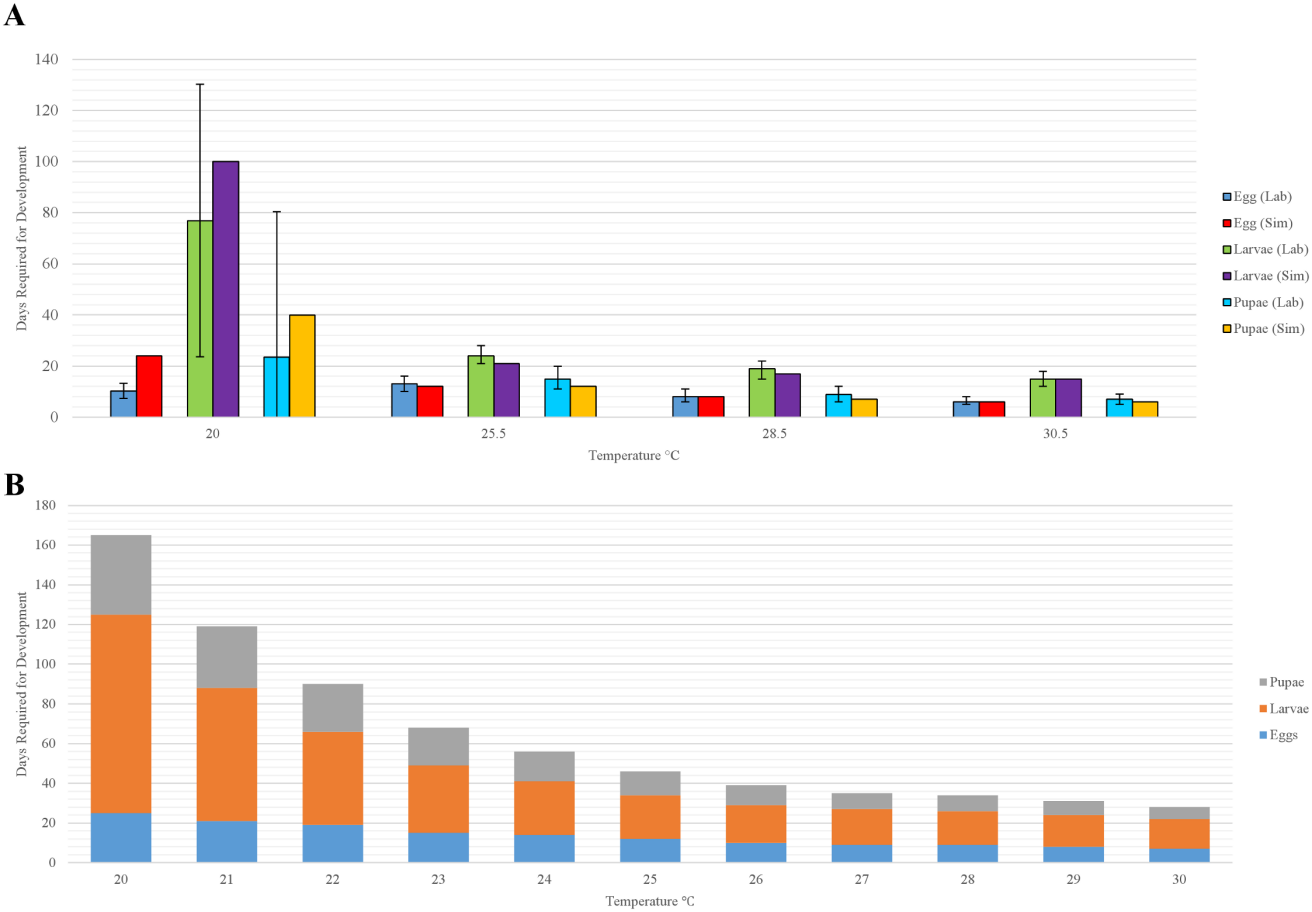
(A) Comparison of simulated and observed mean development times of sand fly eggs, larvae, and pupae at the indicated temperatures, and (B) simulated development times of eggs, larvae, and pupae at the indicated temperatures encompassing the range of soil temperatures collected during a field study in West Bengal, India [49] to which we assumed immature sand flies were exposed. Vertical bars in (A) represent ±1 standard deviation of development times at 20°C [16,18] and the range of development times at temperatures 25.5–30.5°C.

Simulated development times of eggs, larvae, and pupae were similar to those observed by [16,18], except that simulated development times were longer at 20 C (Fig 6a). However, the variability associated with observed development times of larvae and pupae were large. Development times simulated at temperatures encompassing the entire range of temperatures at which laboratory experiments were conducted captured the expected non-linear increase at cooler temperatures [16–18,20], with the total development time of immature stages at 30 C nearly 130 days longer that at 20 C (Fig 6b).

#### Development of adults: Pre-reproduction, pre-oviposition

**Fig 7:**
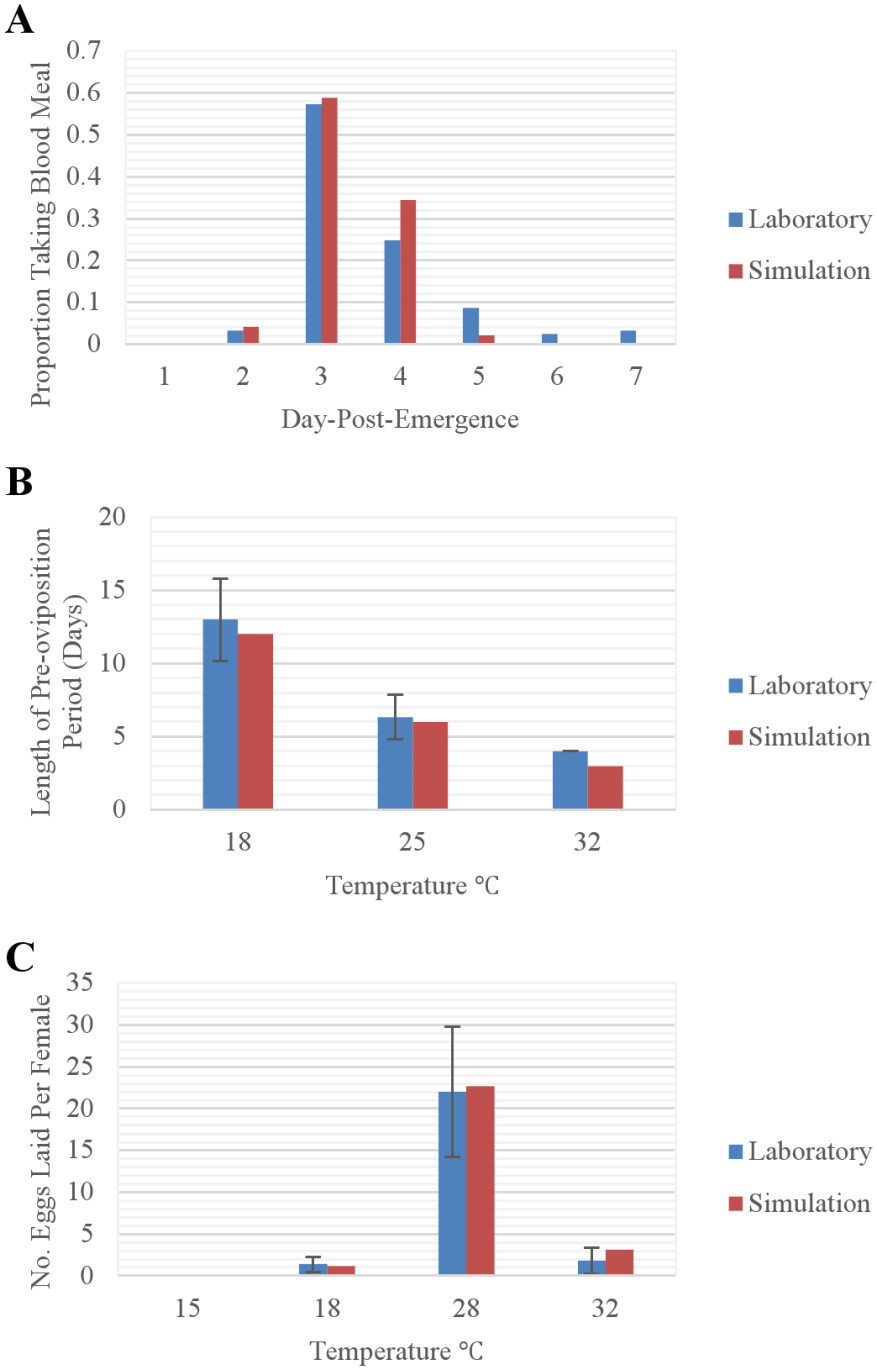
Comparison of (A) simulated and observed daily proportion of blood meals obtained [44] by pre-reproductive females as a function of days-post-emergence, (B) simulated and observed [19] mean lengths of the pre-oviposition period at the indicated temperatures, and (C) simulated and observed [19] number of (female) eggs laid per female at the indicated temperatures. Vertical bars represent ±1 standard deviation. The number of eggs observed in the laboratory in part **c** is divided by two to represent only females.

Simulated lengths of the pre-reproductive stage were similar to those observed by [44], with the majority of simulated sand flies taking blood meals 3 or 4 days-post-emergence (Fig 7a). The simulated proportion of ovipositing females that obtained a second blood meal was similar to that reported by [17] (0.100 versus 0.093). Simulated lengths of the pre-oviposition stage were similar to those observed by [19], with lengths decreasing in response to increasing temperatures (Fig 7b).

#### Reproduction: Eggs laid per reproductive female

Simulated numbers of eggs laid per female were similar to those observed by [19], with by far the highest number of eggs-per-female at 28 C (Fig 7c).

#### Natural mortality

**Fig 8:**
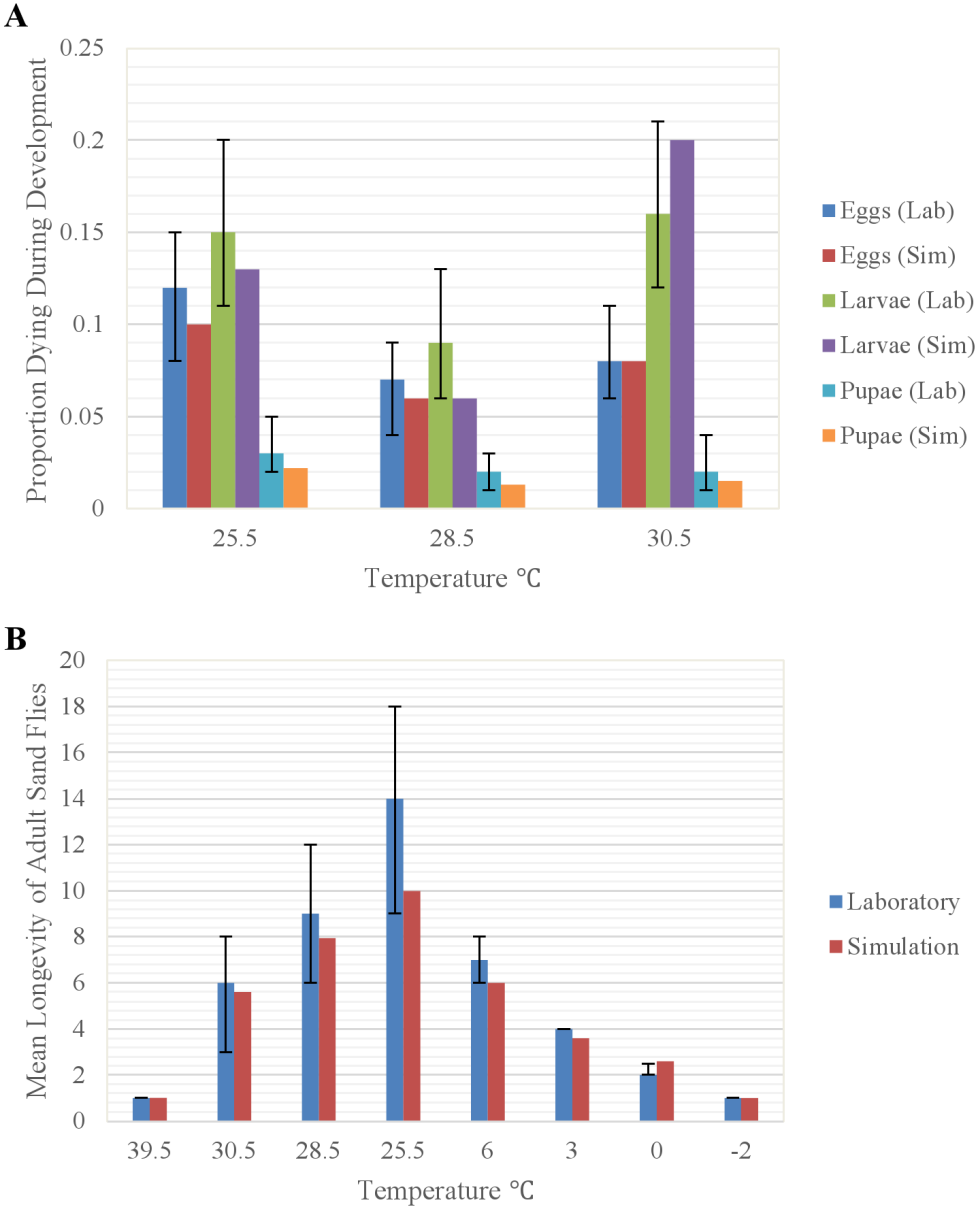
Comparison of (A) simulated and observed [16] natural mortality rates of eggs, larvae, and pupae at the indicated temperatures (range reported), and (B) simulated and observed [16,45] mean longevities of adult sand flies at the indicated temperatures. Vertical bars represent the range of values.

Simulated rates of natural mortality of eggs, larvae, and pupae were similar to those observed by [16], with mortality lowest at 28.5 C and increasing at both cooler and warmer temperatures (Fig 8a). Simulated longevities of adults were similar to those observed by [16,45], with longevity increasing as temperatures decrease from 39.5 to 25.5 C and longevity decreasing as temperatures decrease from 6 to -2 C (Fig 8b).

#### Fipronil-induced mortality

**Fig 9:**
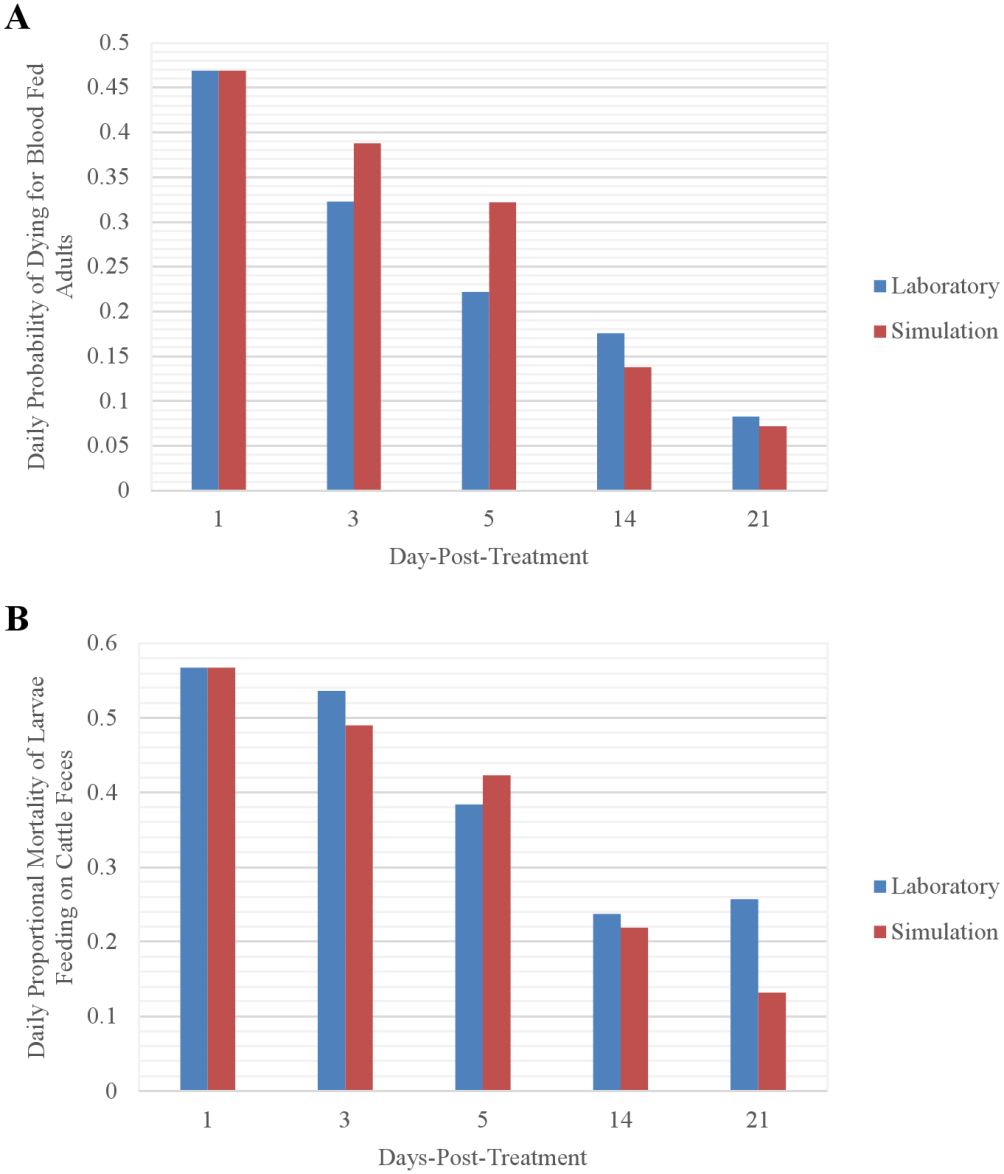
Comparison of (A) simulated and observed [25] probabilities of fipronil-induced mortality of adults obtaining a blood meal the indicated number of days post-treatment, and (B) simulated and observed [25] fipronil-induced mortality rates of larvae exposed to organic matter containing cattle feces deposited the indicated number of days post-treatment.

Simulated probabilities of fipronil-induced mortality of adults were similar to those observed by [25], with the daily probability of mortality decreasing from 0.469 for adults obtaining a blood meal on the day the cow was treated to 0.072 for those obtaining a blood meal 21 days after treatment (Fig 9a). With one exception, simulated rates of fipronil-induced mortality of larvae were similar to those observed by [25], with the daily mortality rate decreasing from 0.567 to 0.132 for larvae exposed to organic matter containing cattle feces deposited from 1 to 21 days post-fipronil-application, respectively (Fig 9b). The exception was that the 21-days post-application mortality rate in the laboratory was greater than the 14-days post-application mortality rate, whereas the simulated mortality rate continued to decrease from 14 to 21 days-post-application.

### Model calibration

**Fig 10:**
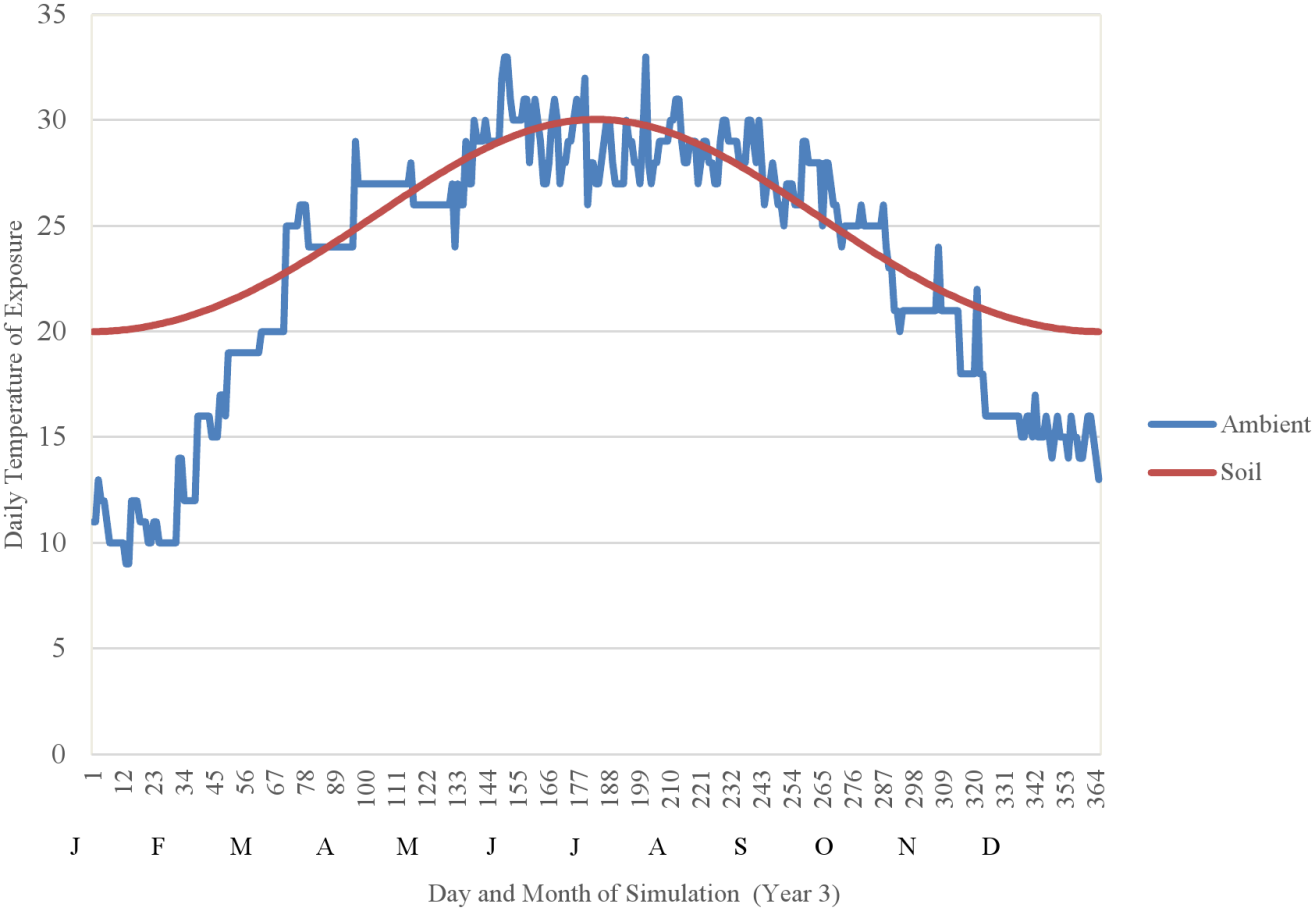
The daily minimum air temperature data recorded at a village in Bihar, India (unpublished data from daily collections used to develop Table 1 in [21]) and a cosine curve fitted to a graphical representation of annual fluctuations in soil temperatures in West Bengal, India [49], which were used to calibrate the time series of air temperatures and temperatures within organic matter, respectively, used in the simulation model.

We calibrated the model to represent environmental conditions typical of Bihar, India by representing annual fluctuations in (1) simulated air temperatures (*T*_*i*,*A*_) with a time series of 365 minimum daily air temperatures recorded at a village in Bihar [21] and (2) simulated temperatures within organic matter (*T*_*i*,*O*_) by fitting a cosine curve to a graphical representation of annual fluctuations in soil temperatures in West Bengal, India presented by [49] (Fig 10). We further calibrated the model by adjusting the parameter controlling the density-dependent mortality of larvae due to cannibalism (9.5 x 10^−7^) such that the simulated population established a seasonally-varying, dynamic equilibrium under baseline conditions (without fipronil-induced mortality) in which the mean annual abundance of adults was approximately equal to the breeding site capacity, or number of vectors (7,344) estimated by [29].

### Model evaluation

**Fig 11:**
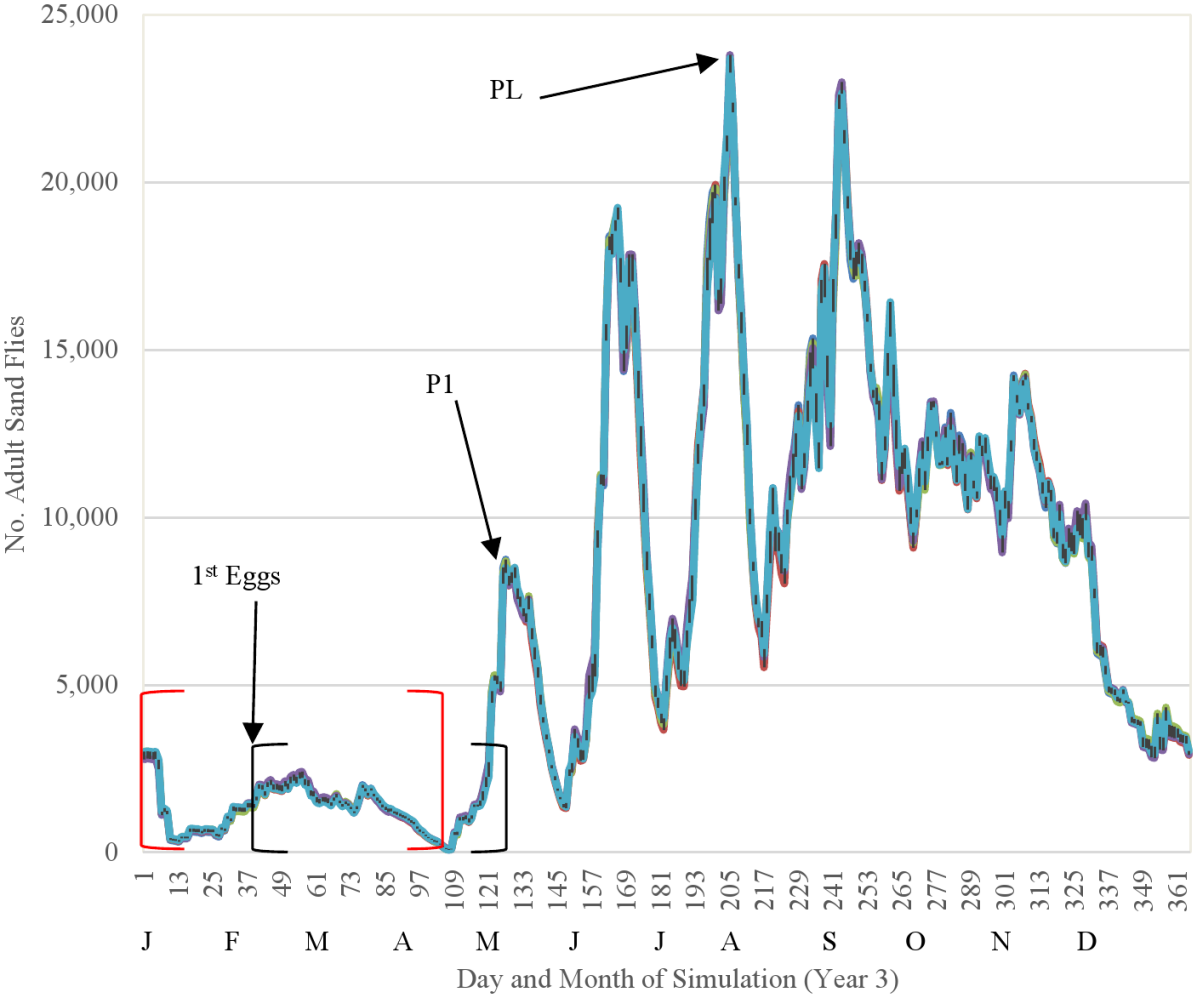
Fluctuations in abundance of adult sand flies observed during year 3 of the baseline simulation. Red brackets indicate a generation of overwintering sand flies. Black brackets indicate the time between initial oviposition and the first post-winter peak in abundance of adults (P1). PL indicates the largest peak abundance of adults.

**Fig 12:**
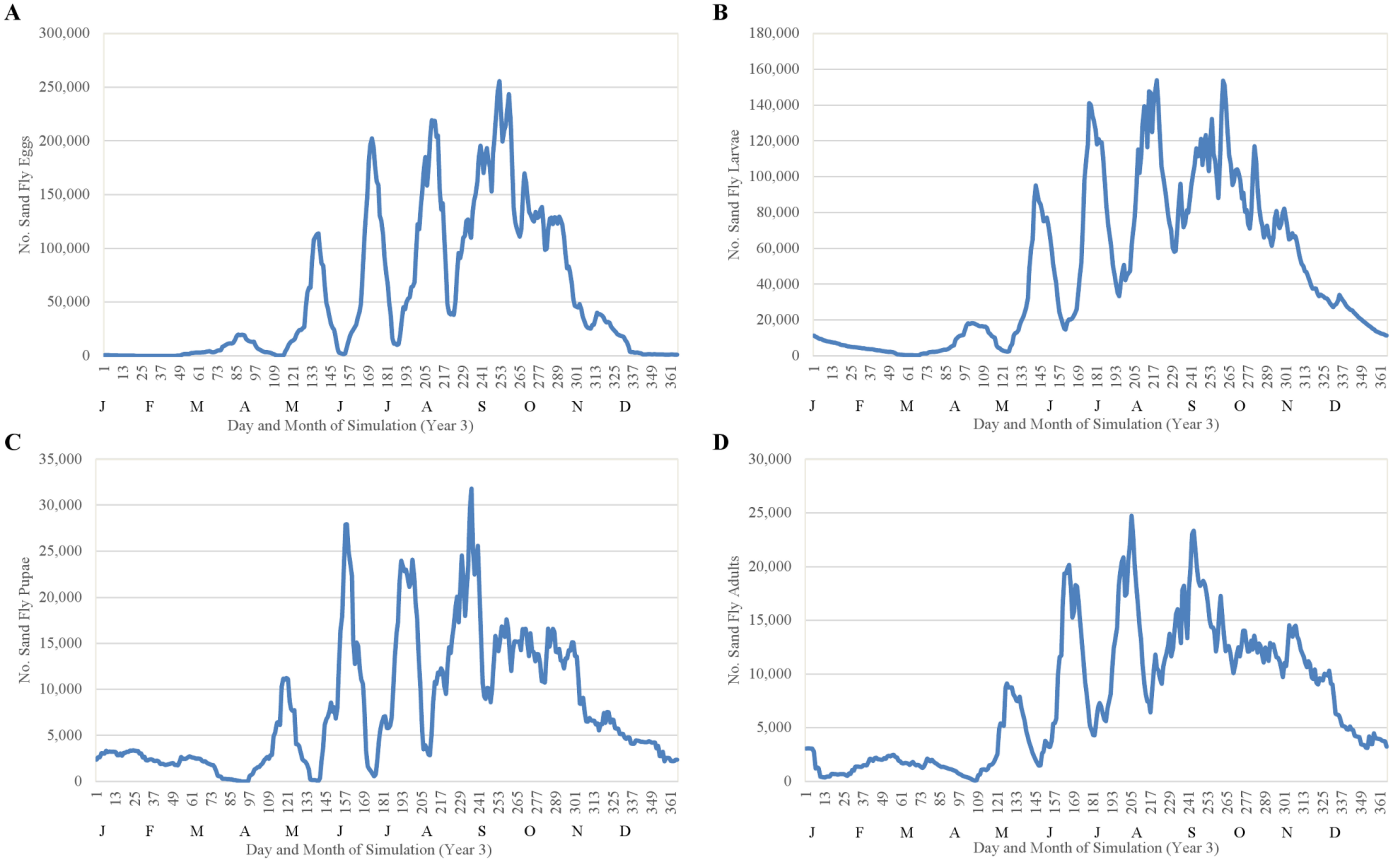
Fluctuations in abundances of (A) eggs, (B) larvae, (C) pupae, and (D) adults observed during year 3 of the baseline simulation.

We evaluated the baseline model by (1) assessing the ecological interpretability of seasonal trends in the simulated sand fly life cycle and (2) comparing simulated fluctuations in relative abundance of adult sand flies to fluctuations in relative abundance of adults caught in light traps in each of three villages in Bihar over a 12-month period using a Sign Test. Simulated seasonal trends were representative of the general temperature-dependent trends characteristic of the sand fly life cycle in Bihar (Fig 11). Simulated oviposition did not occur until mid-February (day-of-year 42), when temperatures first exceeded the 15 C threshold suggested by [19], with the first mass emergence of adults occurring 85 days later during May (day-of-year 127), and the largest peak in adult abundance occurring during the latter portion of July (day-of-year 205), as observed by [21]. Simulated egg, larval, and pupal fluctuations were impossible to validate due to lack of field data, but showed similarity to simulated adult fluctuations (Fig 12a–12d). The fluctuations of egg and pupal population densities were more distinct because the developmental periods are considerably shorter than that of larvae and adults.

**Fig 13:**
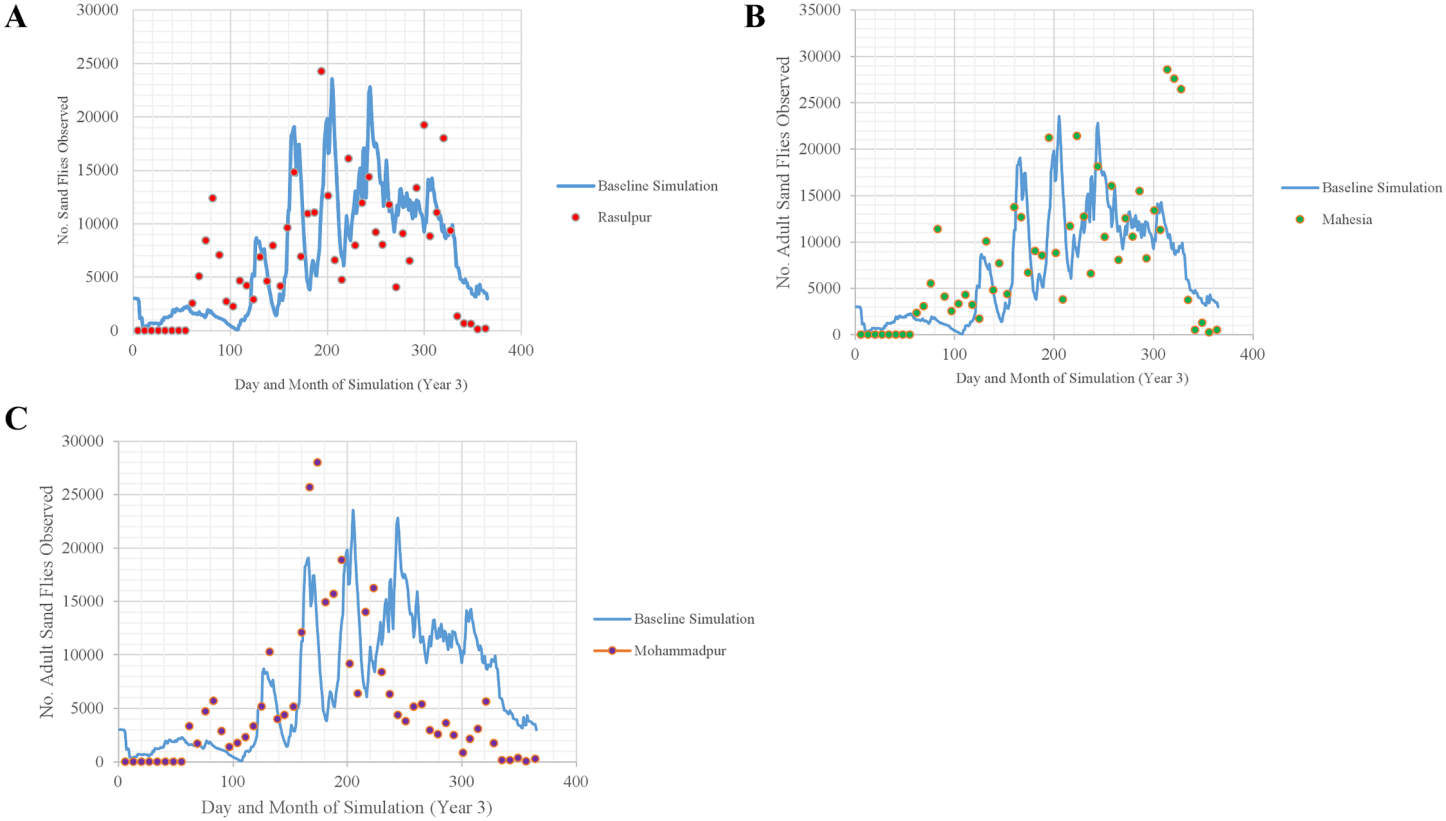
Seasonal abundances of adult sand flies observed during year 3 of the baseline simulation (solid line) and relative numbers of adults caught in light-traps in A) Rasulpur, B) Mahesia, C) Mohammadpur, three villages in Bihar, India (dots, unpublished data from weekly collections used to develop figures and tables in [21]). Field data were scaled (Rasulpur data x 500; Mahesia data x 280; Mohammadpur data x 450) to facilitate comparison of relative seasonal abundances.

Simulated fluctuations in relative abundance of adults were not significantly different from the general trends in relative abundance of adults caught in the three villages in Bihar (sign test: *p* < 0.1263, *p* < 0.5000, and *p* < 0.0704, respectively), although, not surprisingly, trends in the field samples were less distinct [21] (Fig 13). Interestingly, trends in relative abundance at one village (Mohammadpur) were markedly different from both the simulated trends and the trends observed at the other two villages (*p* < 0.10), most likely due to markedly lower abundances during September, October, and November.

### Simulated responses of sand fly populations to control schemes using fipronil-treated cattle

#### Simulation results

**Fig 14:**
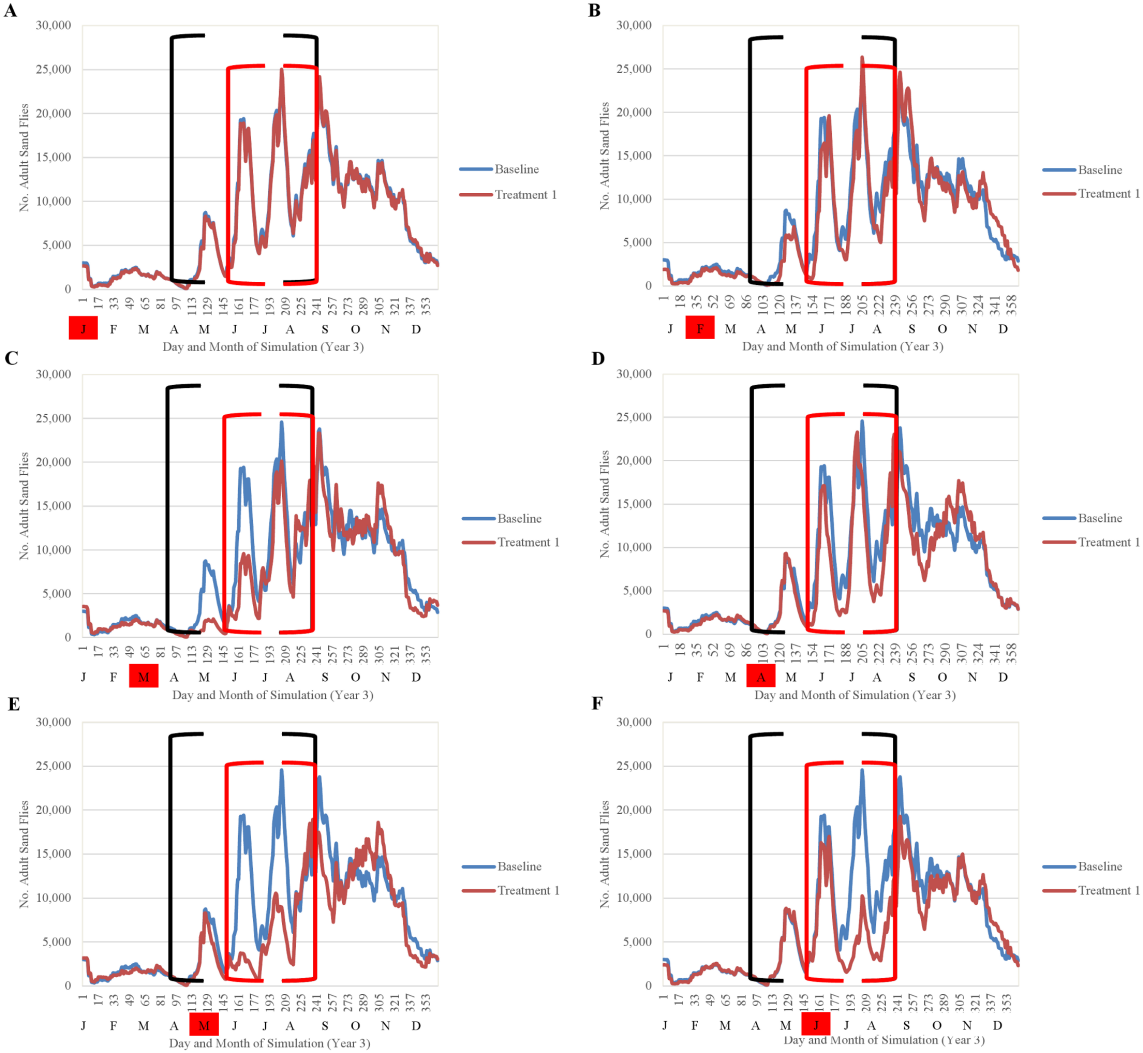
Comparison of the seasonality of adult sand flies observed during year 3 of simulation for treatments performed once annually January-June (A-F) and the baseline simulation (no treatment) (mean 10 replications). Black brackets indicate April-August and the red brackets indicate the summer months of peak human exposure (June-August). Red boxes indicate the months of treatment application.

**Fig 15:**
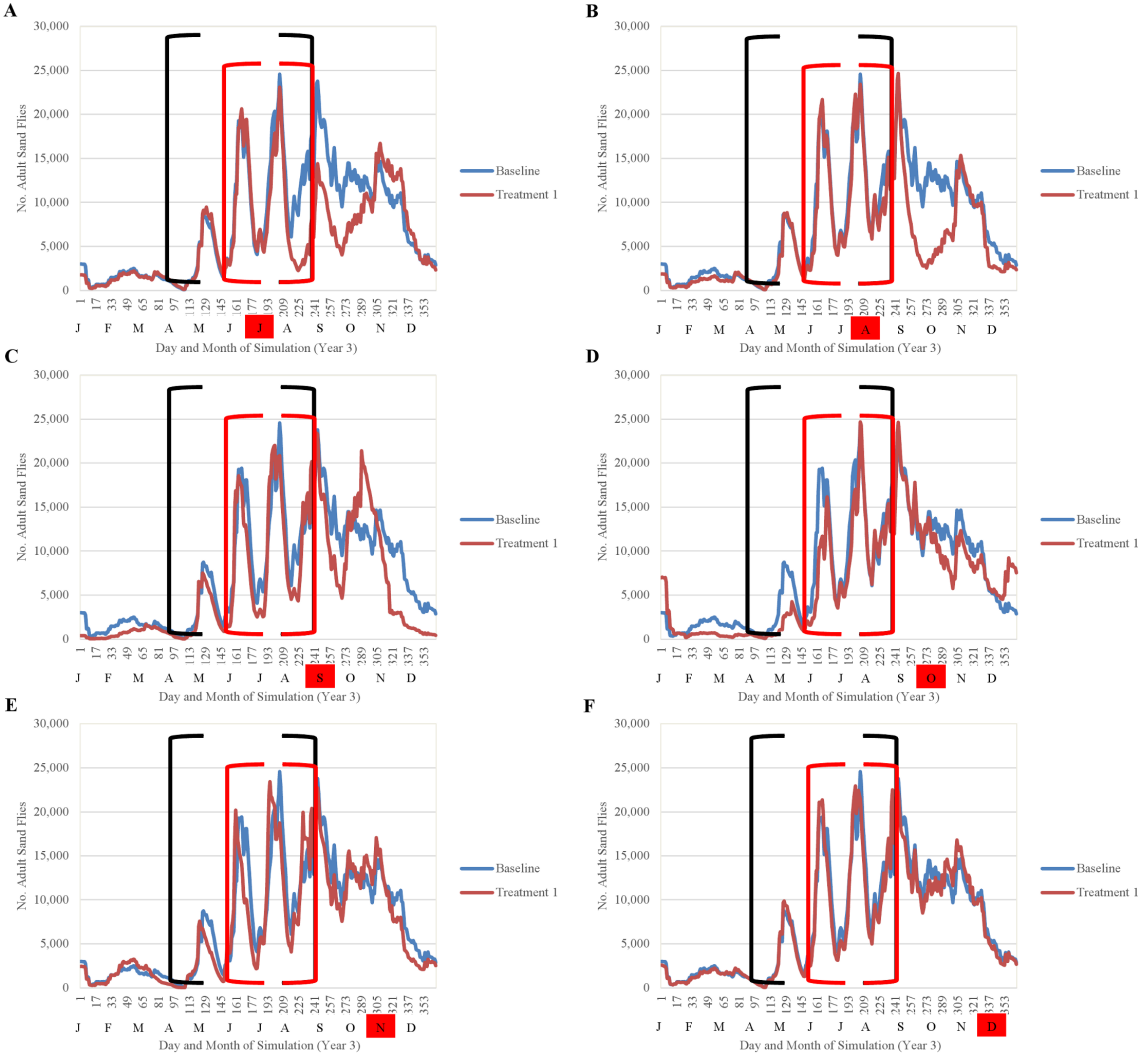
Comparison of the seasonality of adult sand flies observed during year 3 of simulation for treatments performed once annually July-December (A-F) and the baseline simulation (no treatment) (mean 10 replications). Black brackets indicate April-August and the red brackets indicate the summer months of peak human exposure (June-August). Red boxes indicate the months of treatment application.

**Fig 16:**
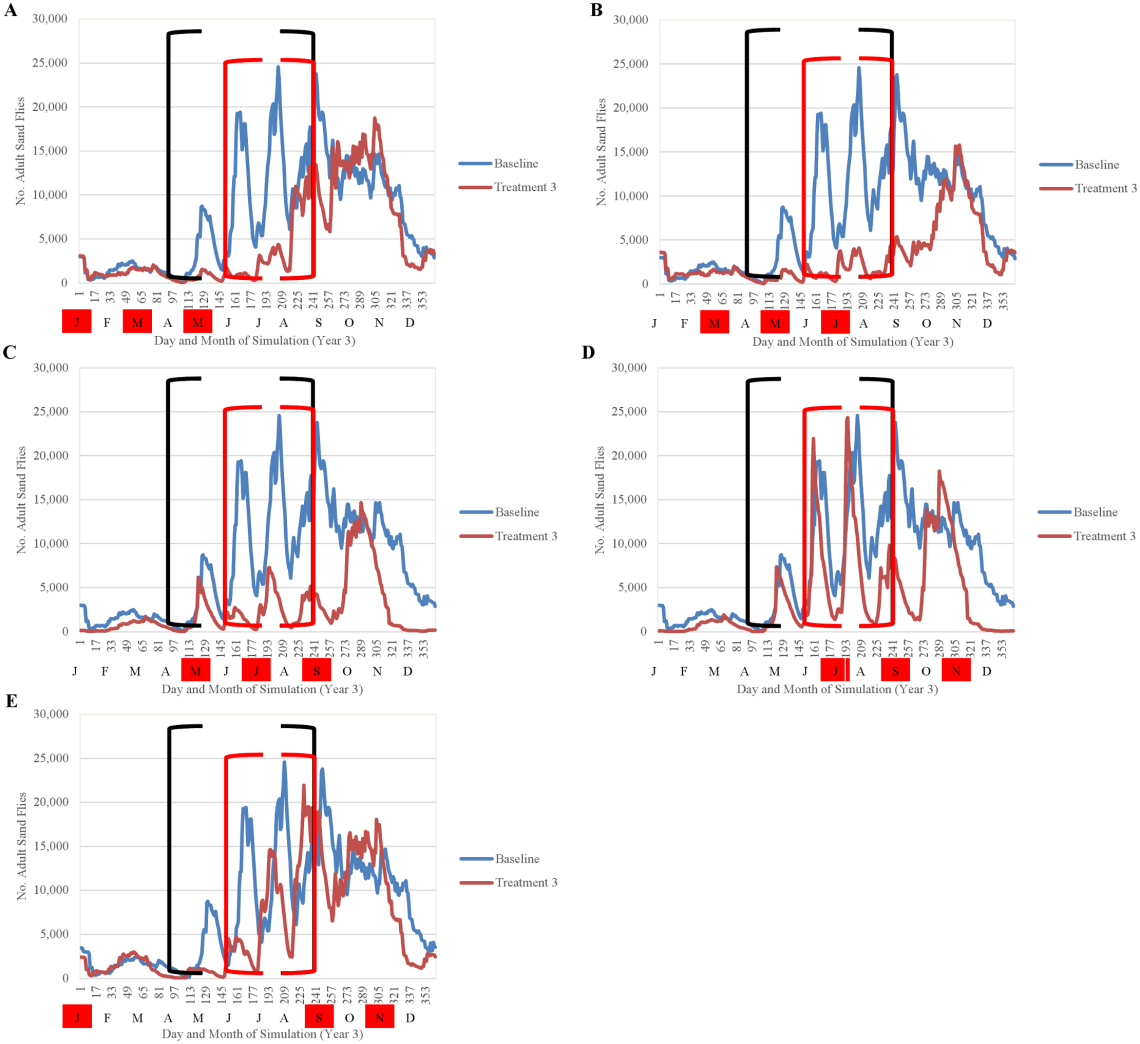
Comparison of the seasonality of adult sand flies observed during year 3 of simulation for treatments performed three times per year (A-E) and the baseline simulation (no treatment) (mean 10 replications). Black brackets indicate April-August and the red brackets indicate the summer months of peak human exposure (June-August). Red boxes indicate the months of treatment application.

**Fig 17:**
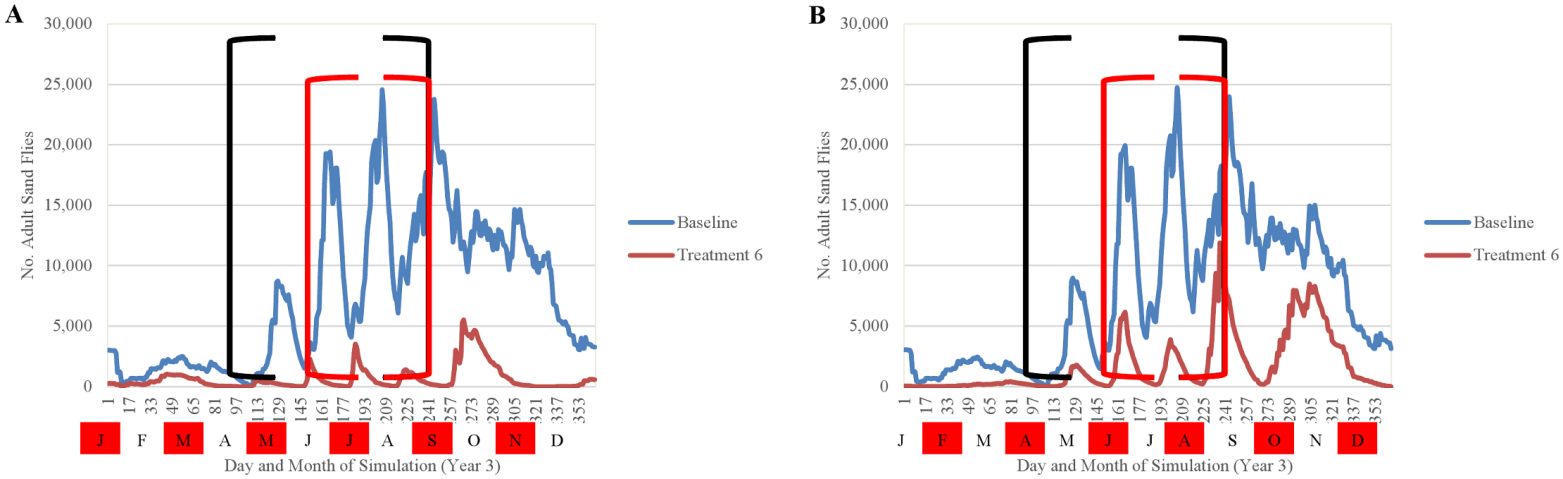
Comparison of the seasonality of adult sand flies observed during year 3 of simulation for treatments performed six times per year (A and B) and the baseline simulation (no treatment) (mean 10 replications). Black brackets indicate April-August and the red brackets indicate the summer months of peak human exposure (June-August). Red boxes indicate the months of treatment application.

**Fig 18:**
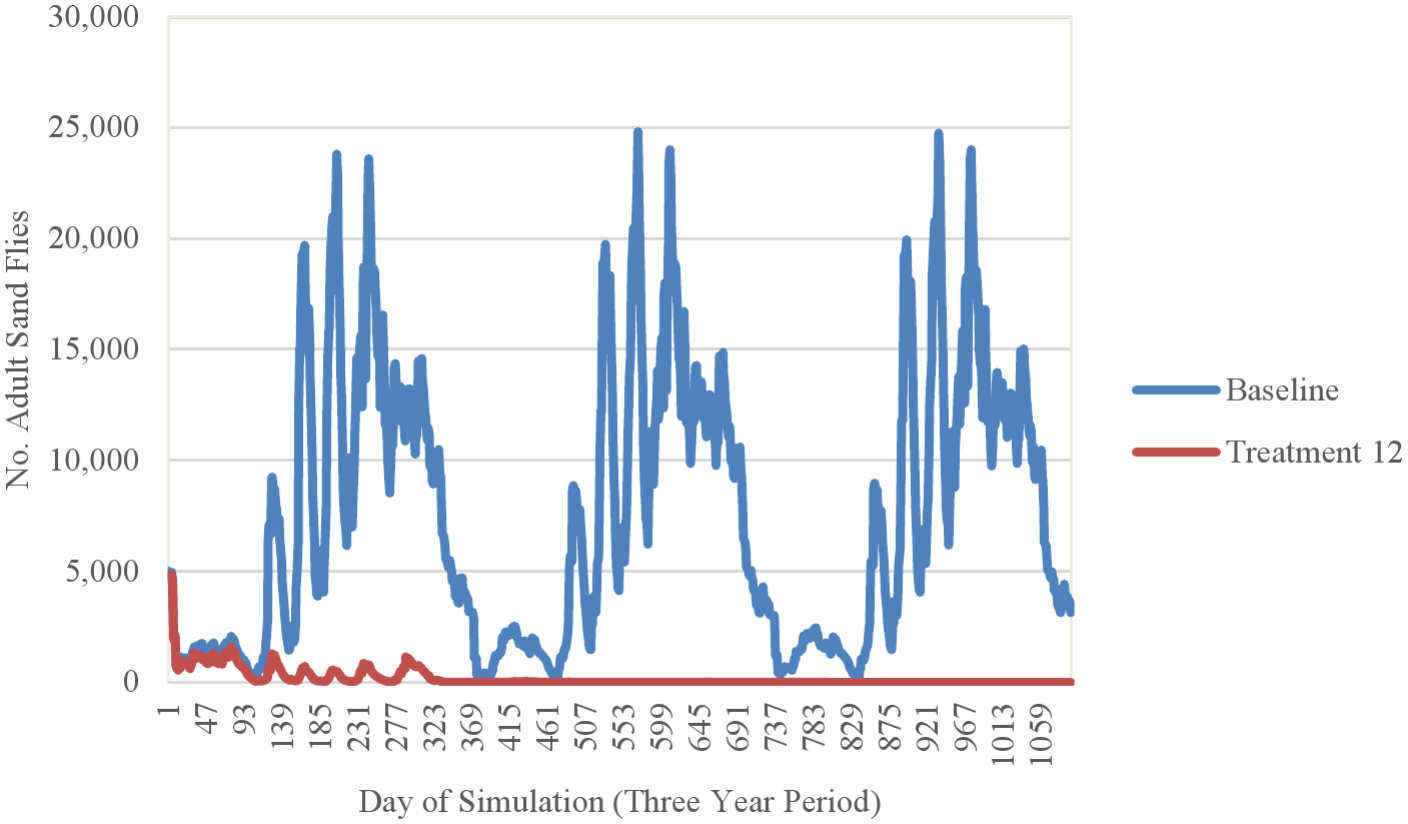
Comparison of the seasonality of adult sand flies observed during a 3-year simulation period for treatments performed 12 times per year and the baseline simulation (no treatment) (mean 10 replications).

Simulation results indicated that the efficacy of fipronil-based control schemes in reducing sand fly abundance depended not only on the frequency of treatment applications, but also on the timing of applications relative to the seasonality of the sand fly life cycle (Figs 14–18). Single annual treatments applied in March, May, June, or July noticeably reduced the population peaks that occurred over the 30 to 60 days following treatment, but populations recovered relatively quickly (Figs 14c, 14e, 14f and 15a). Single annual treatments applied in May produced the most noticeable reduction, with the population peaks occurring in June and July being reduced by ≈80% and ≈60%, respectively, relative to those produced by the baseline (no control) simulation (Fig 14e). Control schemes involving treatments applied 3 times per year at 2-month intervals were most effective when initiated in March, reducing the population peaks occurring from April through August by ≈90% relative to baseline (Fig 16b). Control schemes involving treatments applied 6 times per year at 2-month intervals were most effective when initiated in January, reducing population peaks occurring from June through August by >95% relative to baseline (Fig 17a). The control scheme involving treatments applied 12 times per year at monthly intervals resulted in eradication of the sand fly population within 2 years (Fig 18).

**Fig 19:**
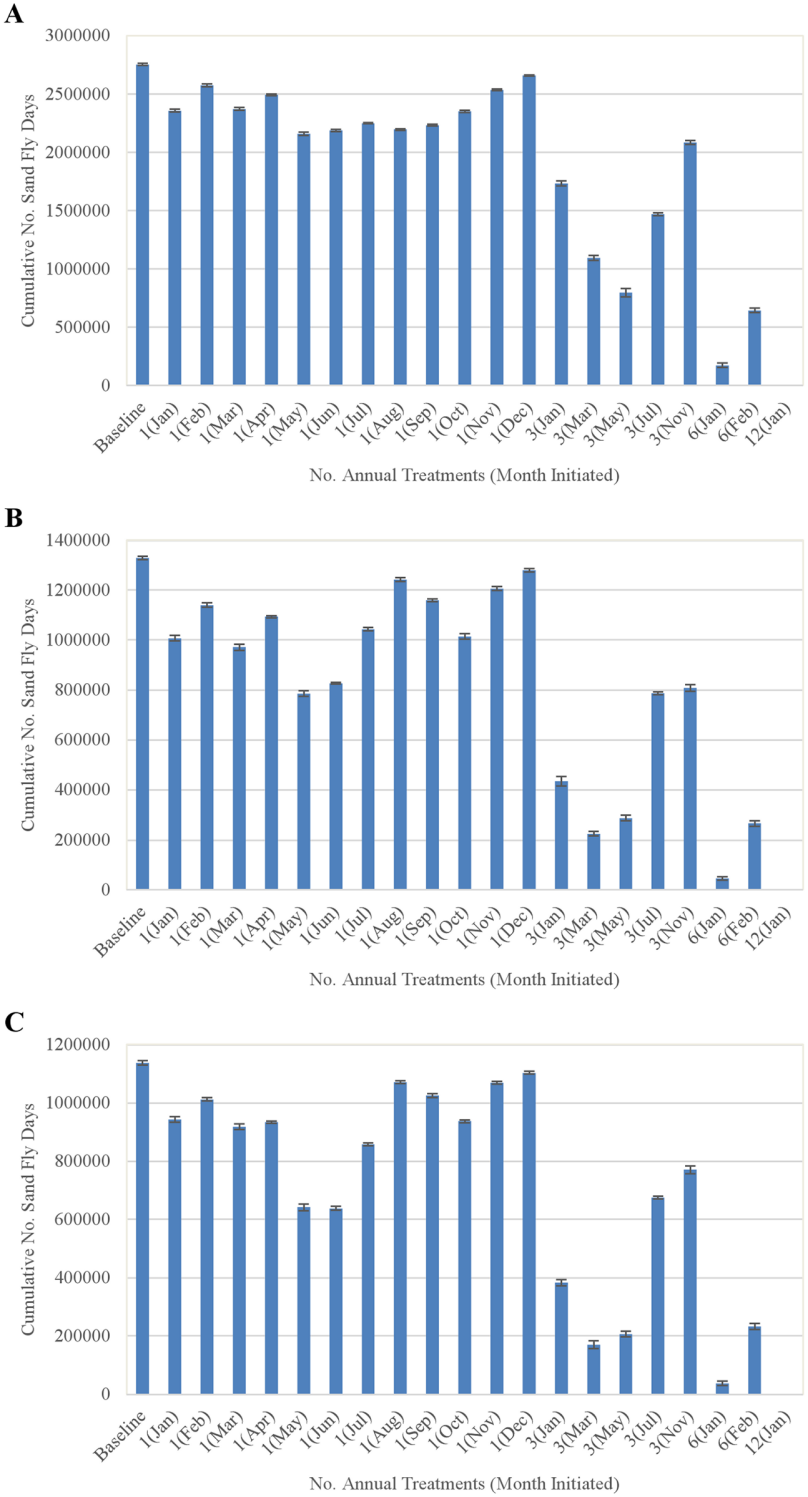
Comparison of (A) the cumulative number of sand fly days over the entire third year of treatment, (B) the cumulative number of sand fly days from April-August of the third year, and (C) the cumulative number of sand fly days from June-August of the third year observed during the indicated treatment scenarios. The x-axis labels indicate the number of treatments per year and the months in parentheses indicate the month of the first treatment each year. Bars represent means (±1 standard error) of 10 replications and vertical arrows of the same color indicate pairs of control schemes that were not significantly different from one another based on Fisher’s least significant difference (LSD) tests. All other pairwise LSD comparisons between control schemes indicated significant (*p* < 0.0001) differences.

Simulation results regarding the efficacy of fipronil-based control schemes in reducing the cumulative number of sand fly days (SFD) occurring annually, occurring during April-August, and occurring specifically during the summer period of peak human exposure (June-August) are summarized in Fig 19. Results of Fisher’s least significant difference (LSD) tests indicated significant (*p* < 0.0001) differences if SFD for the vast majority of pairwise comparisons between control schemes (the exceptions are indicated by vertical arrows in Fig 19). Single annual treatments applied in May reduced SFD by ≈22%, ≈41%, and ≈44%, respectively, during these three periods relative to those produced by the baseline (no control) simulation. Treatments applied 3 times per year at 2-month intervals initiated in March, which included an application in May, reduced SFD by ≈60%, ≈83%, and ≈85%, respectively, during these periods relative to baseline. Treatments applied 6 times per year at 2-month intervals initiated in January, also including a May application, reduced SFD by ≈94%, ≈97%, and ≈97%, respectively, during these periods relative to baseline. Treatments applied 12 times per year at monthly intervals, as mentioned above, resulted in population eradication within 2 years. Thus a treatment application in May was a commonality among all of the more efficacious schemes.

**Fig 20:**
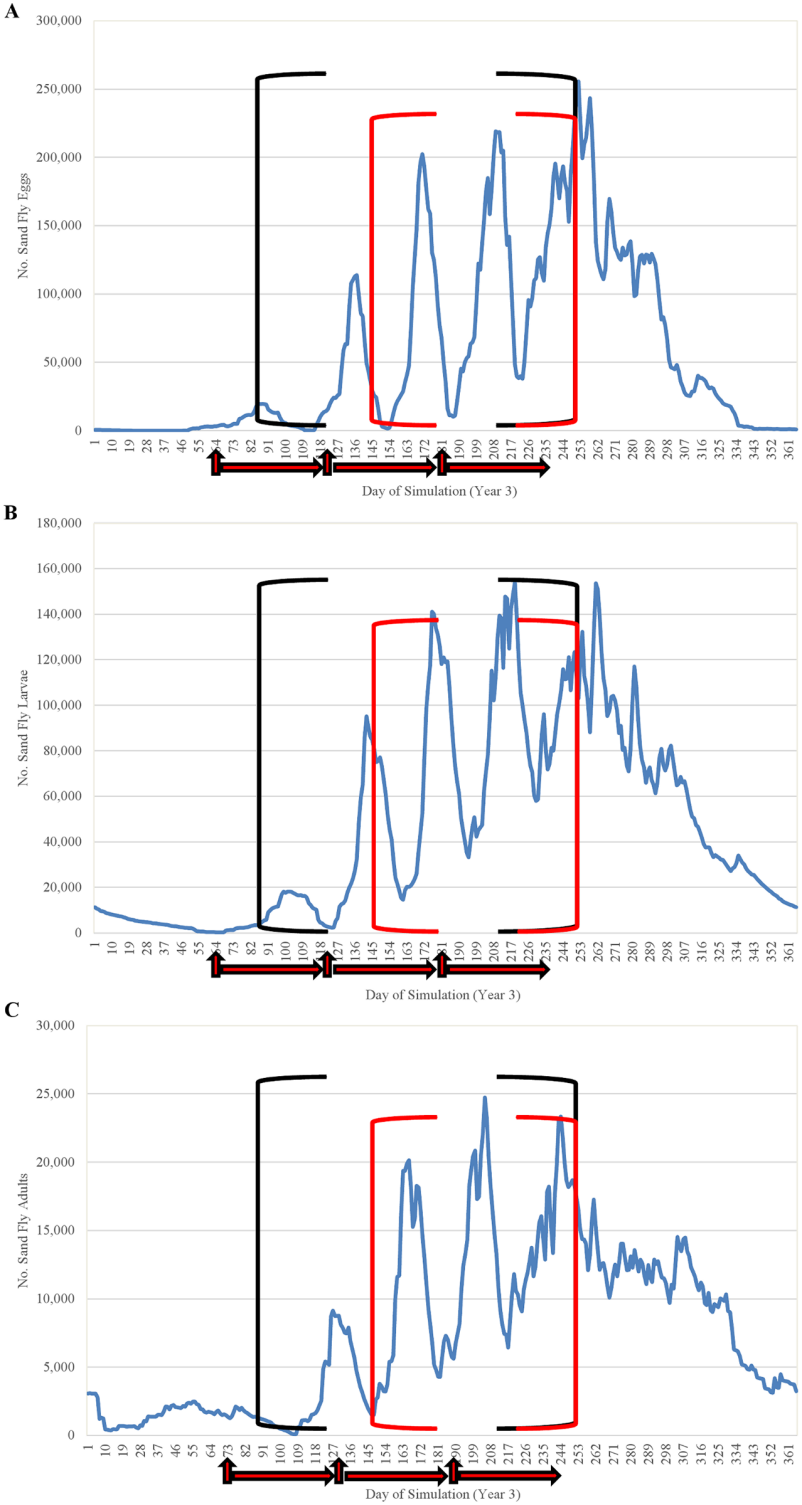
Comparison of the seasonality of (A) eggs, (B) larvae, and (C) adult sand flies observed during year 3 of the baseline simulation (no treatment) to the period during which fipronil efficacy is maintained when treatment is applied three times per year (March, May, July). Vertical red arrows indicate day of application and horizontal red arrows indicate duration of fipronil efficacy (~60 days). Black brackets indicate April-August and the red brackets indicate the summer months of peak human exposure (June-August).

Closer examination of the simulation of the most efficacious scheme involving 3 treatments per year (3 treatments at 2-month intervals initiated in March) provides insight into the processes linking the frequency and timing of treatment applications to the seasonality of the sand fly life cycle (Fig 20). In this scheme, the May application was preceded by an application in March and followed by an application in July, resulting in the most pronounced and prolonged reduction in the sand fly population. Although some oviposition began in mid-February, the first noticeable increase in larval abundance began during March and the first larval peak occurred during April, with the resulting first peak in adult abundance occurring during late April and early May. The three subsequent large summer cohorts passed through their larval stage during May, June, July, and August, feeding in cattle feces containing fipronil which was deposited within the 60-day period following one of the treatment applications (which is the duration of fipronil efficacy in cattle). Thus the relatively high efficacy of this treatment scheme was due to the maintenance of the efficacy of the drug in cattle feces during of period of peak use by larvae, which also included the months of April-August and specifically the period of peak human exposure (outdoor sleeping) from June-August.

Interestingly, with a single annual application in May, as well as with some other treatments in which the drug was administered one or three times a year, the abundance of adult sand flies actually exceeded baseline (non-treated) levels later in the year, usually during October or November (Figs 14–16). This counter-intuitive result was caused by the reduced effect of density-dependent cannibalism among larvae feeding at lower (than baseline) densities in cattle feces in which the efficacy of the drug had diminished markedly since the last treatment application. Thus more (than baseline) larvae pupated and subsequently developed into adults.

An important caveat associated with these results is related to the uncertainty with which we estimated the two parameters representing the proportion of adult sand flies that feed on cattle and the proportion of larvae that feed in organic matter containing cattle feces. Obviously, avoidance of the drug nullifies its effect. Analysis of the sensitivity of predictions of SFD accumulated during June through August under the most efficacious control scheme (treatments applied 3 times per year at 2-month intervals initiated in March) indicated that decreasing the proportion (X) of adult sand flies that fed on cattle from 1 to 0 resulted in an approximately 5-fold increase in SFD from ≈61,000 to ≈306,000 (SFD = 429.95X^2^ + 19497X + 41258, R^2^ = 0.9997). Decreasing the proportion of larvae that fed in organic matter containing cattle feces from 1 to 0 resulted in an approximately 18-fold increase in SFD from ≈54,000 to ≈964,000 (SFD = -4830.4X^2^ + 149729X − 99269, R^2^ = 0.9997).

## Discussion

Simulation results suggest that the success of fipronil treatments in controlling sand flies depends not only on the frequency of applications but also on the timing of applications relative to the sand fly lifecycle. Synchronizing applications to maintain high efficacy of the drug in cattle feces during the period of high larval abundance seems particularly important. While more frequent applications obviously are more efficacious, they also are more expensive and more difficult logistically. Thus, the ability to assess not only efficacy of treatment schemes *per se* but also their cost-effectiveness and their logistical feasibility is of paramount importance. Adequate *a priori* assessment of novel control schemes targeted at specific aspects of a vector’s life cycle requires novel approaches, including models that explicitly represent key aspects of the processes by which the control method intervenes in the life cycle of the target species.

Several previous studies of VL epidemiology have focused on villages in Bihar and have included models with detailed representations of disease dynamics within human populations [29,39–41]. One study modeled the effect of specifically-targeted sand fly control strategies including the application of adulticides and the destruction of breeding sites, which were represented by reducing sand fly life expectancy and breeding site capacity, respectively [29]. Their model predicted that either reducing vector density >67% through application of adulticides or >79% through breeding site destruction could eliminate the ability of the pathogen to persist, as indicated by the value of the basic reproduction number (R_o_ < 1.0; [42]). Although providing a wealth of details concerning disease dynamics within humans and valuable information regarding the general magnitude of vector reduction required to control transmission to humans, environmental factors affecting the vector life cycle, and hence the transmission process, were not modeled explicitly [29]. These authors recognized the limitations this imposed on use of their model and provided appropriate caveats [29]. Other studies also have modeled the effect on VL control via direct manipulation of model parameters controlling mortality rate [50,51] or biting rate [52] of adult sand flies, again providing valuable information pertinent to objectives of their studies, but without explicit representation of environmental factors affecting the seasonality of such rates.

By explicitly representing the effects of seasonally-varying temperatures on development and survival of the various sand fly life stages, our model has allowed initial assessment of a novel control scheme targeted specifically at both larvae and adults. By specifically examining the relationship among the timing and frequency of treatment applications, the duration of drug efficacy, and the seasonality of the sand fly lifecycle, we can make initial assessments not only in terms of reducing average sand fly abundance, but also in terms of cost-effective reduction of human exposure to sand flies given local social practice and availability of alternative hosts.

As a common social practice, some family members of the vast majority of Bihari villager households sleep outdoors, particularly during the months with the hottest evening temperatures (June, July, August) [21,24]. Although indoor residual spraying, when properly applied [22], and bed net usage [31] offer protection against infected sand flies feeding indoors, outdoor sleeping places a sizeable portion of the population at risk of exposure to infected exophilic sand flies during periods of peak sand fly abundance. Thus reduction of sand fly abundance during the period when villagers are most likely to be exposed to outdoor-feeding sand flies is of particular importance when assessing the efficacy of control schemes. Considering the results from our model in terms of the efficacy of the various treatment schemes to reduce sand fly abundance during this critical period.

Economically, Bihar is the poorest state in India, with roughly $100 million gross domestic product compared to the national average of $274 million [53], and people within the VL-endemic zones are among the most impoverished people in the world [54]. Thus considerations of cost effectiveness become paramount in terms of the commercial feasibility of drug application by villagers in VL-endemic regions. The cost of treating with this form of drug is estimated at approximately $1 per cow per treatment, but milk production per cow is estimated to increase by $0.50 per day [55], offering incentive to villagers to pay to treat their animals. Our simulations assumed 100% of cattle were treated, as would occur in a field trial. However, likely <100% of cattle would be treated if this form of treatment became commercially available, since individual livestock owners would be responsible for its application. In this regard, the influence of regional differences in socio-economic conditions on the efficacy of alternative sand fly control schemes should be evaluated closely. Although beyond the scope of the present study, a future use of our model could include a sensitivity analysis aimed at assessing the impact of regional socio-economic differences on the efficacy of different treatment schemes under different hypotheses regarding local economic conditions and social practice.

Potential environmental and human health impacts, as well as effects on non-target species, always are a concern when evaluating new vector control methods. In this regard, treating cattle orally with fipronil-based drugs may have benefits over conventional IRS. IRS often involves the application of insecticides to the walls of homes and cattle sheds, thus exposing human inhabitants as well as non-target species coming into contact with the walls. DDT has known environmental consequences, but chronic exposure also could potentially be linked to human health concerns such as pancreatic cancer [56–58] making the choice to switch to synthetic pyrethroids logical. Fipronil-based drugs can be safely administered to cattle, given the acute oral LD₅₀ for fipronil-fed rats is ≈97mg/kg of body weight [28], and exposure of cattle to orally applied drugs with fipronil at a concentration of 0.5 mg/kg of body weight can provide effective control of adult and larval sand flies. We are not suggesting treatment with fipronil-based drugs as a replacement for IRS, but rather as a complimentary component. The impact of current practices of IRS and bed net administration on the vector population and human VL transmission has been inconclusive and thus there is much interest in alternative control methods and integrated control schemes [22]. By targeting sand flies feeding on cattle outdoors and larvae developing in cattle feces (areas not targeted by IRS), fipronil-based drug treatment may prove to be a potentially important component of an integrated pest management program.

Further evaluation of the effects of sand fly control through the use of fipronil-based drugs orally administered to cattle ideally would involve a field trial in Bihar. Among the most critical data obtained from such an experiment in terms of increasing confidence in our model predictions would be those shedding light on the proportion of adult sand flies that obtain their blood meal from cattle and the proportion of eggs oviposited in organic matter containing cattle feces. By far the most restrictive assumptions we have made in our model are that 50% of adult sand flies obtain their blood meal from cattle [9] and that 90% of sand fly eggs are laid on, and hence larvae develop in, organic matter containing cattle feces [13]. Although it would have been desirable to use sand fly field collections [25] and blood meal analysis of field caught sand flies [9] to parameterize equations representing adult and larval sand fly developmental processes, currently this is infeasible. Locations of immature sand flies in the field are largely unknown, making field observations difficult. It is also difficult to ascertain the age at which blood fed sand flies collected from the field acquire a blood meal. In contrast, daily immature *P*. *argentipes* and *P*. *papatasi* processes and adult blood feeding probability and oviposition can be observed under controlled conditions in the laboratory [16–20, 44].

The interaction of vector feeding tendencies and host availability on the success of vector control based on systemic insecticides is a topic of current investigation. A recent study modeled the effect of assuming different hypothetical functional relationships between biting behavior of mosquitos (e.g., indiscriminate, anthropophilic, zoophilic) and human host availability on subsequent predictions of malarial infections [59]. This author’s model results indicated that control efficacy was dependent on both intrinsic host preferences and variation in encounter rates with alternative hosts. Our model results assumed that a fixed 50% of sand flies blood fed on cattle treated with fipronil and that the remaining 50% fed on alternative hosts that did not contain fipronil in their blood. Previous blood meal analysis suggests opportunistic feeding behavior of *P*. *argentipes*. Research conducted in eight districts in the West Bengal, a state neighboring Bihar, by [10] and in a single district by [12] suggested that *P*. *argentipes* blood feeding was not driven by particular host preference but rather by host availability, as they fed primarily on humans in human dwellings and cattle in cattle sheds. Research conducted by [60] suggests *P*.*argentipes* feed on cattle primarily, but feed readily on humans in human dwellings, with the researchers referring to *P*. *argentipes* as “chance feeders.” In our model, the probability of a sand fly acquiring a blood meal from cattle implicitly represents relative host availability. The results of our sensitivity analysis in which the mean number of SFD decreased in response to an increased proportion of adults feeding on cattle are indicative of the predicted tendency of indiscriminately feeding mosquitoes presented by [59]. It goes without saying that this treatment is dependent on the presence of cattle. Although *P*. *argentipes* feed indiscriminately, they have been reported to be zoophilic in the past as well [11]. Therefore, explicit representation of sand fly feeding tendencies, not unlike that presented by [59], may be a beneficial addition to our model in the future.

Empirical evidence regarding the substrates in which oviposition occurs is sparse. Sensitivity analysis indicated the obvious importance of assumptions which directly affect the exposure of simulated sand flies to the drug. Immature sand flies are typically collected from the floors of cattle sheds and human dwellings in India, but often in small numbers [13–15]. Studies have uncovered sand fly larvae in larger numbers from diverse locations such as forest floors [61] and abandoned sheds [62–64], and adult sand flies have been collected from palm tree canopies in Bihar [23], suggesting oviposition may be occurring in a variety of microhabitats.

Other data limitations affecting parameterization of the present model included the need to use developmental data from laboratory studies of another species of Phlebotomus rather than field data on our target species. The use of *P*. *papatasi*, in addition to *P*. *argentipes*, data for model parameterization was necessary because laboratory data for *P*. *papatasi* are more abundant and the lifetables developed for them [18–19] are from studies conducted under a wider range of temperatures than those of *P*. *argentipes* [16]. The *P*. *papatasi* data provide upper and lower thermal limits [45], and thus provide at least a general basis for predicting how phlebotomine sand flies function at extreme temperatures. Although, similarities have been observed between these two species in terms of development [17], we suggest that laboratory studies be conducted to further study the temperature-dependence of the incriminated vector of VL on the Indian subcontinent, *P*. *argentipes*.

Notwithstanding the inevitable parametric uncertainties associated with the current model, we would suggest that our model structure might be adapted for initial evaluation of fipronil-based sand fly control under a range of different environmental conditions involving a variety of potential hosts. For instance, the VL vector in East Africa, *Phlebotomus orientalis*, is highly zoophilic in Ethiopia but feeds heavily on donkeys in addition to cattle [65,66], suggesting the need to include multiple host species. The hare is a potential reservoir for VL in Spain [67], suggesting a different route of administration may be required, such as a fipronil-based pour-on or a grain bait, rather than a bolus. Dogs are the primary reservoir for zoonotic VL in the Americas [68] and insecticide impregnated dog collars have shown promise in reducing the VL infection rate in dogs [69,70], suggesting that fipronil impregnated dog collars could provide a means of targeting sand flies in Latin America. Although the host species treated and route of drug administration would need to be modified depending on the situation, the current structure of our model should accommodate the necessary re-parameterizations.

While 90% of reported VL cases occur in six countries on the Indian Subcontinent: India, Bangladesh, South Sudan, Sudan, Ethiopia, and Brazil [71], both VL and cutaneous leishmaniasis (CL) also are present in Europe and climatic projections suggest that the Central European climate will become increasingly suitable for sand flies capable of vectoring VL and CL [72]. The large number of refugees fleeing to Europe from countries such as Afghanistan and Iraq, where CL is known to occur and where clinical VL cases have been documented [73], creates the potential for widespread leishmaniasis outbreaks. Our hope is that our model will prove useful in the *a priori* evaluation of the potential role of treatment schemes involving the use of fipronil-based drugs in the control of leishmaniasis on the Indian Subcontinent and beyond.

## Supporting Information

S1 Table Time series of minimum daily air temperatures in Bihar, India monitored from October 2009 through September 2010 as part of the study described in [21]. (DOCX)

S2 Table Total number of sand flies collected per night in three villages in Bihar, India from 28 October 2009 through 20 October 2010. Details of the sampling procedure can be found in [21].
(DOCX)
