# Supplementary material for: Visceral Leishmaniasis on the Indian Subcontinent: Modelling the Dynamic Relationship between Vector Control Schemes and Vector Life Cycles
Source: PLoS Negl Trop Dis. 2016 Aug 18;10(8):e0004868. doi: 10.1371/journal.pntd.0004868 (PMC4990243; doi:10.1371/journal.pntd.0004868)
Supplement: S1 Table — (DOCX) [file pntd.0004868.s001.docx]

| Minimum Air Temperature (℃) | | | | | | | | | | | | |
| --- | --- | --- | --- | --- | --- | --- | --- | --- | --- | --- | --- | --- |
| Day of Month | Month of Year | | | | | | | | | | | |
|  | Jan. | Feb. | Mar. | Apr. | May | Jun. | Jul. | Aug. | Sep. | Oct. | Nov. | Dec. |
| 1 | 11 | 14 | 19 | 24 | 26 | 31 | 28 | 31 | 26 | 25 | 21 | 16 |
| 2 | 11 | 14 | 19 | 24 | 26 | 30 | 27 | 29 | 27 | 25 | 21 | 16 |
| 3 | 13 | 12 | 20 | 24 | 26 | 30 | 27 | 28 | 27 | 25 | 24 | 15 |
| 4 | 12 | 12 | 20 | 24 | 26 | 30 | 28 | 28 | 28 | 25 | 21 | 15 |
| 5 | 12 | 12 | 20 | 24 | 26 | 30 | 29 | 29 | 27 | 25 | 21 | 16 |
| 6 | 11 | 12 | 20 | 29 | 26 | 31 | 30 | 29 | 26 | 26 | 21 | 16 |
| 7 | 10 | 12 | 20 | 27 | 26 | 31 | 30 | 29 | 26 | 25 | 21 | 15 |
| 8 | 10 | 16 | 20 | 27 | 26 | 28 | 28 | 27 | 25 | 25 | 21 | 17 |
| 9 | 10 | 16 | 20 | 27 | 26 | 30 | 27 | 28 | 27 | 25 | 21 | 15 |
| 10 | 10 | 16 | 20 | 27 | 26 | 31 | 27 | 29 | 27 | 25 | 21 | 15 |
| 11 | 10 | 16 | 20 | 27 | 27 | 30 | 27 | 29 | 27 | 25 | 18 | 15 |
| 12 | 10 | 16 | 25 | 27 | 24 | 29 | 27 | 28 | 26 | 25 | 18 | 16 |
| 13 | 9 | 15 | 25 | 27 | 27 | 27 | 30 | 28 | 26 | 25 | 18 | 15 |
| 14 | 9 | 15 | 25 | 27 | 26 | 27 | 29 | 27 | 26 | 26 | 18 | 14 |
| 15 | 12 | 15 | 25 | 27 | 26 | 28 | 29 | 27 | 29 | 24 | 18 | 15 |
| 16 | 12 | 17 | 25 | 27 | 29 | 30 | 28 | 29 | 29 | 23 | 18 | 16 |
| 17 | 12 | 17 | 26 | 27 | 27 | 31 | 28 | 30 | 28 | 23 | 22 | 15 |
| 18 | 11 | 16 | 26 | 27 | 27 | 30 | 27 | 30 | 28 | 21 | 18 | 15 |
| 19 | 11 | 19 | 26 | 27 | 30 | 27 | 29 | 29 | 28 | 21 | 18 | 15 |
| 20 | 11 | 19 | 24 | 27 | 29 | 28 | 33 | 29 | 28 | 20 | 16 | 14 |
| 21 | 10 | 19 | 24 | 27 | 29 | 28 | 28 | 29 | 28 | 21 | 16 | 16 |
| 22 | 10 | 19 | 24 | 27 | 29 | 29 | 27 | 29 | 25 | 21 | 16 | 15 |
| 23 | 11 | 19 | 24 | 27 | 30 | 29 | 28 | 28 | 28 | 21 | 16 | 15 |
| 24 | 11 | 19 | 24 | 27 | 29 | 30 | 28 | 28 | 28 | 21 | 16 | 14 |
| 25 | 10 | 19 | 24 | 27 | 29 | 31 | 29 | 28 | 27 | 21 | 16 | 14 |
| 26 | 10 | 19 | 24 | 28 | 29 | 30 | 29 | 30 | 26 | 21 | 16 | 15 |
| 27 | 10 | 19 | 24 | 26 | 29 | 30 | 29 | 30 | 26 | 21 | 16 | 16 |
| 28 | 10 | 19 | 24 | 26 | 29 | 32 | 29 | 29 | 25 | 21 | 16 | 16 |
| 29 | 10 | NA | 24 | 26 | 32 | 26 | 30 | 28 | 24 | 21 | 16 | 15 |
| 30 | 10 | NA | 24 | 26 | 33 | 28 | 30 | 30 | 25 | 21 | 16 | 14 |
| 31 | 10 | NA | 24 | NA | 33 | NA | 31 | 28 | NA | 21 | NA | 13 |
