# Supplementary material for: Visceral Leishmaniasis on the Indian Subcontinent: Modelling the Dynamic Relationship between Vector Control Schemes and Vector Life Cycles
Source: PLoS Negl Trop Dis. 2016 Aug 18;10(8):e0004868. doi: 10.1371/journal.pntd.0004868 (PMC4990243; doi:10.1371/journal.pntd.0004868)
Supplement: S2 Table — Details of the sampling procedure can be found in [21]. (DOCX) [file pntd.0004868.s002.docx]

| Month | Day | Total Sand Flies Collected | | |
| --- | --- | --- | --- | --- |
|  |  | Rasulpur | Mahesia | Mohammadpur |
| January | 6 | 0 | 0 | 0 |
| January | 13 | 0 | 0 | 0 |
| January | 20 | 0 | 0 | 0 |
| January | 27 | 0 | 0 | 0 |
| February | 3 | 3 | 1 | 0 |
| February | 10 | 4 | 9 | 2 |
| February | 17 | 6 | 3 | 0 |
| February | 24 | 9 | 7 | 8 |
| March | 3 | 107 | 176 | 155 |
| March | 10 | 213 | 231 | 79 |
| March | 17 | 354 | 414 | 220 |
| March | 24 | 520 | 854 | 266 |
| March | 31 | 297 | 308 | 134 |
| April | 7 | 114 | 190 | 64 |
| April | 14 | 95 | 250 | 82 |
| April | 21 | 196 | 322 | 107 |
| April | 28 | 176 | 241 | 155 |
| May | 5 | 122 | 129 | 241 |
| May | 12 | 289 | 754 | 480 |
| May | 19 | 194 | 360 | 186 |
| May | 26 | 334 | 578 | 204 |
| June | 2 | 175 | 327 | 240 |
| June | 9 | 404 | 1032 | 565 |
| June | 16 | 622 | 950 | 1199 |
| June | 23 | 291 | 501 | 1307 |
| June | 30 | 460 | 677 | 696 |
| July | 7 | 464 | 641 | 733 |
| July | 14 | 1019 | 1592 | 881 |
| July | 21 | 530 | 660 | 428 |
| July | 28 | 277 | 285 | 298 |
| August | 4 | 199 | 877 | 653 |
| August | 11 | 676 | 1607 | 758 |
| August | 18 | 335 | 956 | 392 |
| August | 25 | 502 | 495 | 295 |
| September | 1 | 604 | 1361 | 204 |
| September | 8 | 387 | 791 | 177 |
| September | 15 | 337 | 1204 | 240 |
| September | 22 | 495 | 604 | 251 |
| September | 29 | 170 | 940 | 138 |
| October | 6 | 381 | 792 | 120 |
| October | 13 | 274 | 1161 | 169 |
| October | 20 | 561 | 617 | 116 |
| October | 28 | 808 | 1004 | 39 |
| November | 3 | 371 | 847 | 99 |
| November | 10 | 464 | 2143 | 143 |
| November | 17 | 756 | 2069 | 263 |
| November | 24 | 393 | 1985 | 81 |
| December | 1 | 56 | 280 | 7 |
| December | 9 | 28 | 39 | 7 |
| December | 16 | 26 | 96 | 16 |
| December | 23 | 5 | 19 | 2 |
| December | 30 | 8 | 39 | 12 |
